# Supplementary material for: Coevolution of the Tlx homeobox gene with medusa development (Cnidaria: Medusozoa)
Source: Commun Biol. 2023 Jul 11;6:709. doi: 10.1038/s42003-023-05077-6 (PMC10336111; doi:10.1038/s42003-023-05077-6)
Supplement: Supplementary file 2 — Supplementary Information [file 42003_2023_5077_MOESM2_ESM.pdf]

Supplementary Information

Supplementary Notes

Supplementary note 1: Alignment used in Figure 1

>TLX1_Homo_sapiens

ISFGIDQILRRIGHPYQNRTPPKKKKPRTSFTRLQICELEKRFHRQKYLASAERAALAKALKMTDAQVKTW
FQNRRTKWRRQT

>TLX1_Mus_musculus

ISFGIDQILRRIGHPYQNRTPPKKKKPRTSFTRLQICELEKRFHRQKYLASAERAALAKALKMTDAQVKTW
FQNRRTKWRRQT

>TLX1_Gallus_gallus

ISFGIDQILRRIGHPYQNRTPPKKKKPRTSFTRLQICELEKRFHRQKYLASAERAALAKALKMTDAQVKTW
FQNRRTKWRRQT

>TLX1_Alligator_mississippiensis

ISFGIDQILRRIGHPYQNRTPPKKKKPRTSFTRLQICELEKRFHRQKYLASAERAALAKALKMTDAQVKTW
FQNRRTKWRRQT

>TLX1_Danio_rerio

ISFGIDQILRRIGHPYQNRTPPKKKKPRTSFTRLQICELEKRFHRQKYLASAERAALAKALKMTDAQVKTW
FQNRRTKWRRQT

>TLX2_Homo_sapiens

ISFGIDQILRRIGHPYQNRTPPKRKKPRTSFSRSQVLELERRFLRQKYLASAERAALAKALRMTDAQVKTW
FQNRRTKWRRQT

>TLX2_Rattus_norvegicus

ISFGIDQILRRIGHPYQNRTPPKRKKPRTSFSRSQVLELERRFLRQKYLASAERAALAKALRMTDAQVKTW
FQNRRTKWRRQT

>TLX2_Monodelphis_domestica

ISFGIDQILRRVGHPYQNRTPPKRKKPRTSFSRAQVLELERRFLRQKYLASAERAALAKALRMTDAQVKT
WFQNRRTKWRRQT

>TLX2_Sarcophilus_harrisii

ISFGIDQILRRVGHPYQNRTPPKRKKPRTSFSRAQVLELERRFLRQKYLASAERAALAKALRMTDAQVKT
WFQNRRTKWRRQT

>TLX2_Equus_asinus

ISFGIDQILRRIGHPYQNRTPPKRKKPRTSFSRSQVLELERRFLRQKYLASAERAALAKALRMTDAQVKTW

FQNRRTKWRRQT

>TLX3_Homo_sapiens

ISFGIDQILRRIGHPYQNRTPPKRKKPRTSFSRVQICELEKRFHRQKYLASAERAALAKSLKMTDAQVKTW
FQNRRTKWRRQT

>TLX3_Rattus_norvegicus

ISFGIDQILRRIGHPYQNRTPPKRKKPRTSFSRVQICELEKRFHRQKYLASAERAALAKSLKMTDAQVKTW
FQNRRTKWRRQT

>TLX3_Gallus_gallus

ISFGIDQILRRIGHPYQNRTPPKRKKPRTSFSRVQICELEKRFHRQKYLASAERAALAKSLKMTDAQVKTW
FQNRRTKWRRQT

>TLX3_Xenopus_laevis

ISFGIDQILRRIGHPYQNRTPPKRKKPRTSFSRVQICELEKRFHRQKYLASAERAALAKSLKMTDAQVKTW
FQNRRTKWRRQT

>TLX3_Danio_rerio

ISFGIDQILRRIGHPYQNRTPPKRKKPRTSFSRVQICELEKRFHRQKYLASAERAALAKTLKMTDAQVKTW
FQNRRTKWRRQT

>TLX3_Monodelphis_domestica

ISFGIDQILRRIGHPYQNRTPPKRKKPRTSFSRVQICELEKRFHRQKYLASAERAALAKSLKMTDAQVKTW
FQNRRTKWRRQT

>TLX3_Python_bivittatus

ISFGIDQILRRIGHPYQNRTPPKRKKPRTSFSRVQICELEKRFHRQKYLASAERAALAKSLKMTDAQVKTW
FQNRRTKWRRQT

>Clytia_hemisphaerica_Tlx

LSFSIERILRRLGHPYTSRASPKRRAFRHTFTPYQVVQLEKLFEQSRYLSSSERMRMSRELKMTDNQLKT
WYQNRRTKLKREI

>Turritopsis_sp_Tlx

LSFSIENILRRIGHPYKSRAPPKRKPCRNTFTQSQIIQLETLFKKTKYLTSAERLHVAKEINMTDSQLKTWY
QNRRTKLKREI

>Scolionema_suvaense_TLX_from_genome

LKFSIENILRRIGHPYQSRAPPKRKPIRHSFTANQVAELEKLFEKSKYLSSSERQRLASELKMTDSQLKTW
YQNRRTKLKREM

>TLX_Craspedacusta_sowerbii_from_genome_ok

LSFSIDSILRRIGHPYQSRAPPKRKPVRHSFTTSQVAQLEKLFEKSKYLSSADRQRVATQLKMTDSQLKT
WYQNRRTKMKREI

>TLX_Podocoryna_carnea

LSFSIESILRRIGHPYQSRAPPKRKPIRNTFTLPQVQKLEQLFHQTKYLSSAERLRVAKELNMTDSQLKTW
YQNRRTKLKREI

>TLX_Aequorea_australis

LSFSIDSILRRIGHPYKSRAPPKRKPCRKTFTQTQVLQLEKLFEQTRYLSSAERLRMAKELNMTDSQLKTW
YQNRRTKLKREL

>TLX_Laodicea_undulata

ISFSIESILRRIGHPYKSRAPPKRKPCRNTFTQTQVFQLEKLFEQNKYLSSAERLRMAKELNMTDNQLKTW
YQNRRTKLKREM

>TLX_Ptychogena_crocea

ISFSIESILRRIGHPYKSRAPPKRKPCRNTFTPTQVIQMEKLFDQTKYLSSAERLRMAKELDMTDNQLKTW
YQNRRTKMKREM

>TLX_Bargmannia_amoena

LSFSIENILRRIGHPYQSRAPPKRKAIRNSFTPNQILHLEKLFEKAKYLPSPERLRVAKELHITDNQLKTWYQ
NRRTKLKREL

>TLX_Bragmannia_elongata

LSFSIENILRRIGHPYQSRAPPKRKAIRNSFTPNQILHLEKLFEKAKYLPSPERLRVAKELHITDNQLKTWYQ
NRRTKLKREL

>TLX_Physophora_gilmeri

LSFSIENILRRIGHPYKSRAPPKRKAVRNSFTPGQILRLEKLFEQTKYLPSPERLRVAKELHITDNQLKTWY
QNRRTKLKREI

>TLX_Apolemia_lanosa

LSFSIENILRRIGHPYQSRAPPKRKAIRNSFSPNQILQLEKLFEQTKYLPSPERLRVANELHITDNQLKTWY
QNRRTKLKREL

>TLX_Apolemia_sp

LSFSIDNILRRIGHPYQSRAPPKRKAIRNSFSPNQILQLEKLFEQTKYLPSPERLRVANELHITDNQLKTWY
QNRRTKLKREL

>TLX_Apolemia_rubriversa

LSFSIENILRRIGHPYQSRAPPKRKATRNSFSPNQILQLEKLFQQTKYLPSPERLRVANELHITDNQLKTWY
QNRRTKLKREL

>TLX_Forskalia_asymmetrica

LSFSIENILRRIGHPYQSRAPPKRKAVRNSFTPSQVLHLEKLFEQTKYLPSPERLRVAKELHITDNQLKTWY
QNRRTKLKREI

>TLX_Lilyopsis_fluoracantha

LSFSIENILRRIGHPYQSRAPPKRKAVRNSFTPSQILHLERLFQQTKYLPSPERLRVAKELRITDNQLKTWY
QNRRTKLKREL

>TLX_Atolla_vanhoeffeni

LSFSIHKILRRIGHPYQSRAPPKRKKPRASFTRSQVTELEKLFIKKKYLTSSERQKVAKSLEMSDCQVKTW
FQNRRTKWKRET

>TLX_Aurelia_aurita

ITFSIRRILRRIGHPYQSRAAPKRKKPRTTFSRSQIAELEELFTEKKYLTSSERQRVANYLTLSDCQVKTWF
QNRRTKWKRET

>TLX_Aurelia_sp1_californian_strain

ISFSINRILRRIGHPYQSRAAPKRKKPRTTFSRSQIAELEELFTEKKYLTSSERQRVANYLSLSDCQVKTWF
QNRRTKWKRET

>TLX_Aurelia_aurita_Kiel_bay_baltic_sea

ISFSISRILRRIGHPYQSRAAPKRKKPRTTFSRSQIAELEELFTEKKYLTSSERQRVANYLDLSDCQVKTWF
QNRRTKWKRET

>TLX_Aurelia_sp_complex_Pacific

ISFSINRILRRIGHPYQSRAAPKRKKPRTTFSRSQIAELEELFTEKKYLTSSERQRVANYLSLSDCQVKTWF
QNRRTKWKRET

>TLX_Aurelia_coerulea

ISFSINRILRRIGHPYQSRAAPKRKKPRTTFSRSQIAELEELFTEKKYLTSSERQRVANYLSLSDCQVKTWF
QNRRTKWKRET

>TLX_Sanderia_malayensis

LSFSIKRILRRIGHPYQSRATPKRKKPRTSFTRSQISELEELFTEKKYLTSSERQRVAACLQLTDCQVKTWF
QNRRTKWKRET

>TLX_Chrysaora_achlyos

LSFSIRRILRRIGHPYQSRATPKRKKPRTTFSRSQISELEELFTEKKYLTSSERQRVANYLQLSDCQVKTW
FQNRRTKWKRET

>TLX_Chrysaora_fuscescens

LSFSIRRILRRIGHPYQSRATPKRKKPRTTFSRSQISELEELFTEKKYLTSSERQRVANYLQLSDCQVKTW
FQNRRTKWKRET

>TLX_Cassiopea_xamachana

LSFSISRILRRIGHPYQSRAVPKRKKPRTTFSRAQITELEDLFTEKKYLTSSERQKVASYLNLSDCQVKTWF
QNRRTKWKRET

>TLX_Rhopilema_esculentum

LSFSISRILRRIGHPYQSRAAPKRKKPRTTFSRAQIAELEELFTEKKYLTSSERQKVASYLNLSDCQVKTWF
QNRRTKWKRET

>TLX_Nemopilema_nomurai

LSFSISRILRRVGHPYQSRAAPKRKKPRTTFSRAQIGELEELFTEKKYLTSSERQKVANYLSLSDCQVKTW
FQNRRTKWKRET

>TLX_Lucernaria_quadricornis

ITFSISRILRRVGHPYQSRAPPKRKRPRKAFESDQVEELEHLFHDKRYLASAERQAVARQLGMTDGQVKT
WFQNRRTKWKRQT

>TLX_Calvadosia_cruxmelitensis

ISFSINRILRRIGHPYQSRAPPRRKQPRKSFEADQVAELEQIFHDKRYLTSGERQNVARQLKMTDNQVKT
WFQNRRTKWKRQF

>TLX_Craterolophus_convolvulus

ISFSINRILRRIGHSYQSRALPYRKQPRKTFESRQVDELELLFHTKRYLTSYERQSVAKQLQMSDSQVKTW
FQNRRTKWKRQC

>TLX_Chironex_yamaguchii

LSFSISRILRRLGHPYQSRAPPKRKKQRASFSRHQIKELEKLFSKKKYLSSSERQQIAKKLDMTDCQVKTW
YQNRRTKWKREK

>TLX_Chironex_fleckeri

LSFSISRILRRLGHPYQSRAPPKRKKQRASFSRNQIKELEKLFSKKKYLSSSERQQIAKKLDMTDCQVKTW
YQNRRTKWKREK

>TLX_Alatina_alata

LSFSISRILRRLGHPYQSRAPPKRKKQRASFSRSQIKELEKLFSKKKYLSSSERQQIAKKLDMTDCQVKTW
YQNRRTKWKREK

>TLX_Morbakka_virulenta

LSFSISRILRRLGHPYQSRAPPKRKKQRASFSRGQIKELEKLFSKKKYLSSSERQQIAKNLDMTDCQVKTW
YQNRRTKWKREN

>TLX_Tripedalia_cystophora

LSFSISRILRRLGHPYQSRAPPKRKKQRASFSRSQIKELEKLFSKKKYLSSSERQHIAKKLDMTDCQVKTW
YQNRRTKWKREK

>TLX_Copula_sivicksi

LSFSISRILRRLGHPYQSRAPPKRKKQRASFSRGQIKELEKLFSKKKYLSSSERQQIAKKLEMTDCQVKTW
YQNRRTKWKREK

>TLX_Carybdea_marsupialis

LSFSISRILRRLGHPYQSRAPPKRKKQRASFSRSQIKELEKLFSKKKYLSSSERQQIAKKLDMTDCQVKTW
YQNRRTKWKREK

>TLX_Alatinidae_sp

LSFSISRILRRLGHPYQSRAPPKRKKQRASFSRSQIKELEKLFSKKKYLSSSERQQIAKKLDMTDCQVKTW
YQNRRTKWKREK

>NK6_Homo_sapiens

KKHSRPTFSGQQIFALEKTFEQTKYLAGPERARLAYSLGMTESQVKVWFQNRRTKWRKRH

>NK6_Danio_rerio

KKHSRPTFSGQQIFALEKTFEQTKYLAGPERARLAYSLGMTESQVKVWFQNRRTKWRKRH

>NK6_Stylophora_pistillata

RKHTRPTFSGHQIFALEKTFEQTKYLAGPERTRLAYSLGMTESQVKVWFQNRRTKWRKRH

>NK6_Exaiptasia_pallida

RKHTRPTFSGHQIFALEKTFEQTKYLAGPERARLAYSLGMTESQVKVWFQNRRTKWRKRH

>NK6_Copula_sivicksi

KKSSRPTFSGHQIYHLERTFEQTKYLAGPERTRLAHIIGMTENQVKVWFQNRRTKWRKKT

>NK6_Ectopleura_larynx

KSTSRATFKGHQVFHLERVFQKKKYLAGPERASLAKLLMLSENQVKVWFQNRRTKWRKKM

>NK6_Turritopsis_sp_SK_2016

KKQSRPTFNGHQIFHLEKTFESTKYLAGPERSRLAKVLHMSENQVKVWFQNRRTKWRKKL

>NK6_Podocoryna_carnea

KKSSRPTFNGHQIFHLEKTFEKTKYLAGPERGRLAKALRMSENQVKVWFQNRRTKWRKKL

>NK6_Hydractinia_symbiolongicarpus

PKSSRPTFNGHQIFHLEKTFEKTKYLAGPERGRLAKALRMSENQVKVWFQNRRTKWRKKL

>NK6_Rhopilema_esculentum

KKSSRPTFNGRQIYHLEKTFEQTKYLAGPERTRLAHYLAMTENQVKVWFQNRRTKWRKKT

>NK6_Aurelia_sp1

KKSSRPTFNGHQIYHLEKTFEQTKYLAGPERTRLAHYLAMTENQVKVWFQNRRTKWRKKT

>NK6_Clytia_hemisphaerica

NKSSRPTFNGHQIFHLEKTFETTKYLAGPERGRLAKALRMSENQVKVWFQNRRTKWRKKL

>Hex_Homo_sapiens

RKGGQVRFSNDQTIELEKKFETQKYLSPPERKRLAKMLQLSERQVKTWFQNRRAKWRRLK

>Hex_Patiria_miniata

RKGGQVRFSNDQTMELEKKFESQKYLSPPERKKLAKLLQLSERQVKTWFQNRRAKWRRVK

>Hex_Danio_rerio

RKGGQVRFSNDQTIELEKKFETQKYLSPPERKRLAKMLQLSERQVKTWFQNRRAKWRRLK

>Hex_Pocillopora_damicornis

KKGGQVRFSNEQTMELEKIFENQKYLSPPERKQLSKVLGLTERQVKTWFQNRRAKWRRFK

>Hex_Porites_astreoides

KKGGQVRFSNEQTLELEKIFESQKYLSPPERKQLSKVLGLTERQVKTWFQNRRAKWRRFK

>Hex_Nematostella_vectensis

KKGGQVRFSNEQTMELEKIFETQKYLSPPERKQLSKVLGLSERQVKTWFQNRRAKWRRFK

>Hex_Stylophora_pistillata

KKGGQVRFSNEQTMELEKIFENQKYLSPPERKQLSKVLGLTERQVKTWFQNRRAKWRRFK

>Hex_Hydractinia_symbiolongicarpus

RKGGQVRFSHTQSSELERVFSIQKYISPQERKQLSRTLDLTERQVKTWFQNRRAKWRRIK

>Hex_Aurelia_aurita

RKGGQVRFSHVQSVELERIFSIQKYISPQERKQLSRFLHLSERQVKTWFQNRRAKWRRIK

>Hex_Clytia_hemisphaerica

RKGGQVRFTHTQSTELERVFSVQKYVSPQERKQLARSIDLSERQVKTWFQNRRAKWRRIK

>Hex_Podocoryna_carnea

RKGGQVRFSHTQSSELERVFSLQKYISPQERKQLSRTLDLTERQVKTWFQNRRAKWRRIK

>Hex_Xenopus_Laevis

RKGGQVRFSNDQTIELEKKFETQKYLSPPERKRLAKMLQLSERQVKTWFQNRRAKWRRLK

>Hex_Amphiura_filiformis

RKGGQVRFSNDQTLELEKKFENQKYLSPPERKKLAKLLQLSERQVKTWFQNRRAKWRRLK

>Hex_Mus_musculus

RKGGQVRFSNDQTVELEKKFETQKYLSPPERKRLAKMLQLSERQVKTWFQNRRAKWRRLK

Supplementary note 2: Alignment used in Figure S1

>TLX1_Homo_sapiens

ISFGIDQILRRIGHPYQNRTPPKKKKPRTSFTRLQICELEKRFHRQKYLASAERAALAKALKMTDAQVKTW
FQNRRTKWRRQT

>TLX1_Mus_musculus

ISFGIDQILRRIGHPYQNRTPPKKKKPRTSFTRLQICELEKRFHRQKYLASAERAALAKALKMTDAQVKTW
FQNRRTKWRRQT

>TLX1_Gallus_gallus

ISFGIDQILRRIGHPYQNRTPPKKKKPRTSFTRLQICELEKRFHRQKYLASAERAALAKALKMTDAQVKTW
FQNRRTKWRRQT

>TLX1_Alligator_mississippiensis

ISFGIDQILRRIGHPYQNRTPPKKKKPRTSFTRLQICELEKRFHRQKYLASAERAALAKALKMTDAQVKTW
FQNRRTKWRRQT

>TLX1_Danio_rerio

ISFGIDQILRRIGHPYQNRTPPKKKKPRTSFTRLQICELEKRFHRQKYLASAERAALAKALKMTDAQVKTW
FQNRRTKWRRQT

>TLX_Crassostrea_gigas

LSFGISRILRRIGHPYQNRTPPKRKKPRTSFSRLQIMELEKRFHRQKYLASAERSTLAKALKMTDAQVKTW
FQNRRTKWRRQT

>TLX_Pecten_maximus

LSFGISRILRRIGHPYQNRTPPKRKKPRTSFTRLQIMELEKRFHRQKYLASAERSALAKALKMTDAQVKTW
FQNRRTKWRRQT

>TLX__Mizuhopecten_yessoensis

LSFGISRILRRIGHPYQNRTPPKRKKPRTSFTRLQIMELEKRFHRQKYLASAERSALAKALKMTDAQVKTW
FQNRRTKWRRQT

>TLX_Glomeris_marginata

LSFSISRLLRRIGHPYQNRTPPKRKKPRTSFTRMQICELEKRFHKQKYLASAERASLAKALKMTDAQVKT
WFQNRRTKWRRQT

>TLX_Centruroides_sculpturatus

LSFSISRLLRRIGHPYQNRTPPKRKKPRTSFTRMQICELEKRFHKQKYLASAERAALAKQLKMTDAQVKT

WFQNRRTKWRRQT

>TLX_Limulus_polyphemus

LSFSISRLLRRIGHPYQNRTPPKRKKPRTSFTRMQICELEKRFHKQYLASAERAALAKQLKMTDAQVKT
WFQNRRTKWRRQT

>TLX_Acanthaster_planci

LSFSISRILRRVGHPYQNRTPPKRKKPRTSFTRLQICELEKRFHRQKYLASAERAGLAKTLKMTDAQVKT
WFQNRRTKWRRQT

>TLX2_Homo_sapiens

ISFGIDQILRRIGHPYQNRTPPKRKKPRTSFSRSQVLELERRFLRQKYLASAERAALAKALRMTDAQVKTW
FQNRRTKWRRQT

>TLX1_Platynereis_dumerilii

LSFGISRILRRIGHPYQNRTPPKRKKPRTSFTRLQIIELEKRFHRQKYLASAERSALAKSLKMTDAQVKTWF
QNRRTKWRRQT

>TLX2_Rattus_norvegicus

ISFGIDQILRRIGHPYQNRTPPKRKKPRTSFSRSQVLELERRFLRQKYLASAERAALAKALRMTDAQVKTW
FQNRRTKWRRQT

>TLX2_Monodelphis_domestica

ISFGIDQILRRVGHPYQNRTPPKRKKPRTSFSRAQVLELERRFLRQKYLASAERAALAKALRMTDAQVKT
WFQNRRTKWRRQT

>TLX2_Sarcophilus_harrisii

ISFGIDQILRRVGHPYQNRTPPKRKKPRTSFSRAQVLELERRFLRQKYLASAERAALAKALRMTDAQVKT
WFQNRRTKWRRQT

>TLX2_Equus_asinus

ISFGIDQILRRIGHPYQNRTPPKRKKPRTSFSRSQVLELERRFLRQKYLASAERAALAKALRMTDAQVKTW
FQNRRTKWRRQT

>TLX3_Homo_sapiens

ISFGIDQILRRIGHPYQNRTPPKRKKPRTSFSRVQICELEKRFHRQKYLASAERAALAKSLKMTDAQVKTW
FQNRRTKWRRQT

>TLX3_Rattus_norvegicus

ISFGIDQILRRIGHPYQNRTPPKRKKPRTSFSRVQICELEKRFHRQKYLASAERAALAKSLKMTDAQVKTW
FQNRRTKWRRQT

>TLX3_Gallus_gallus

ISFGIDQILRRIGHPYQNRTPPKRKKPRTSFSRVQICELEKRFHRQKYLASAERAALAKSLKMTDAQVKTW
FQNRRTKWRRQT

>TLX3_Xenopus_laevis

ISFGIDQILRRIGHPYQNRTPPKRKKPRTSFSRVQICELEKRFHRQKYLASAERAALAKSLKMTDAQVKTW
FQNRRTKWRRQT

>TLX3_Danio_rerio

ISFGIDQILRRIGHPYQNRTPPKRKKPRTSFSRVQICELEKRFHRQKYLASAERAALAKTLKMTDAQVKTW
FQNRRTKWRRQT

>TLX3_Monodelphis_domestica

ISFGIDQILRRIGHPYQNRTPPKRKKPRTSFSRVQICELEKRFHRQKYLASAERAALAKSLKMTDAQVKTW
FQNRRTKWRRQT

>TLX3_Python_bivittatus

ISFGIDQILRRIGHPYQNRTPPKRKKPRTSFSRVQICELEKRFHRQKYLASAERAALAKSLKMTDAQVKTW
FQNRRTKWRRQT

>TLX_Clytia_hemisphaerica

LSFSIERILRRLGHPYTSRASPKRRAFRHTFTPYQVVQLEKLFEQSRYLSSSERMRMSRELKMTDNQLKT
WYQNRRTKLKREI

>TLX_Turritopsis_sp

LSFSIENILRRIGHPYKSRAPPKRKPCRNTFTQSQIIQLETLFKKTKYLTSAERLHVAKEINMTDSQLKTWY
QNRRTKLKREI

>TLX_Scolionema_suvaense

LKFSIENILRRIGHPYQSRAPPKRKPIRHSFTANQVAELEKLFEKSKYLSSSERQRLASELKMTDSQLKTW
YQNRRTKLKREM

>TLX_Craspedacusta_sowerbii

LSFSIDSILRRIGHPYQSRAPPKRKPVRHSFTTSQVAQLEKLFEKSKYLSSADRQRVATQLKMTDSQLKT
WYQNRRTKMKREI

>TLX_Podocoryna_carnea

LSFSIESILRRIGHPYQSRAPPKRKPIRNTFTLPQVQKLEQLFHQTKYLSSAERLRVAKELNMTDSQLKTW
YQNRRTKLKREI

>TLX_Aequorea_australis

LSFSIDSILRRIGHPYKSRAPPKRKPCRKTFTQTQVLQLEKLFEQTRYLSSAERLRMAKELNMTDSQLKTW
YQNRRTKLKREL

>TLX_Laodicea_undulata

ISFSIESILRRIGHPYKSRAPPKRKPCRNTFTQTQVFQLEKLFEQNKYLSSAERLRMAKELNMTDNQLKTW
YQNRRTKLKREM

>TLX_Ptychogena_crocea

ISFSIESILRRIGHPYKSRAPPKRKPCRNTFTPTQVIQMEKLFDQTKYLSSAERLRMAKELDMTDNQLKTW
YQNRRTKMKREM

>TLX_Bargmannia_amoena

LSFSIENILRRIGHPYQSRAPPKRKAIRNSFTPNQILHLEKLFEKAKYLPSPERLRVAKELHITDNQLKTWYQNRRTKLKREL

>TLX_Bragmannia_elongata

LSFSIENILRRIGHPYQSRAPPKRKAIRNSFTPNQILHLEKLFEKAKYLPSPERLRVAKELHITDNQLKTWYQNRRTKLKREL

>TLX_Physophora_gilmeri

LSFSIENILRRIGHPYKSRAPPKRKAVRNSFTPGQILRLEKLFEQTKYLPSPERLRVAKELHITDNQLKTWYQNRRTKLKREI

>TLX_Apolemia_lanosa

LSFSIENILRRIGHPYQSRAPPKRKAIRNSFSPNQILQLEKLFEQTKYLPSPERLRVANELHITDNQLKTWYQNRRTKLKREL

>TLX_Apolemia_sp

LSFSIDNILRRIGHPYQSRAPPKRKAIRNSFSPNQILQLEKLFEQTKYLPSPERLRVANELHITDNQLKTWYQNRRTKLKREL

>TLX_Apolemia_rubriversa

LSFSIENILRRIGHPYQSRAPPKRKATRNSFSPNQILQLEKLFQQTKYLPSPERLRVANELHITDNQLKTWYQNRRTKLKREL

>TLX_Forskalia_asymmetrica

LSFSIENILRRIGHPYQSRAPPKRKAVRNSFTPSQVLHLEKLFEQTKYLPSPERLRVAKELHITDNQLKTWYQNRRTKLKREI

>TLX_Lilyopsis_fluoracantha

LSFSIENILRRIGHPYQSRAPPKRKAVRNSFTPSQILHLERLFQQTKYLPSPERLRVAKELRITDNQLKTWYQNRRTKLKREL

>TLX_Atolla_vanhoeffeni

LSFSIHKILRRIGHPYQSRAPPKRKKPRASFTRSQVTELEKLFIKKKYLTSSERQKVAKSLEMSDCQVKTWFQNRRTKWKRET

>TLX_Aurelia_aurita

ITFSIRRILRRIGHPYQSRAAPKRKKPRTTFSRSQIAELEELFTEKKYLTSSERQRVANYLTLSDCQVKTWFQNRRTKWKRET

>TLX_Aurelia_sp1_californian_strain

ISFSINRILRRIGHPYQSRAAPKRKKPRTTFSRSQIAELEELFTEKKYLTSSERQRVANYLSLSDCQVKTWFQNRRTKWKRET

>TLX_Aurelia_aurita_Kiel_bay_baltic_sea

ISFSISRILRRIGHPYQSRAAPKRKKPRTTFSRSQIAELEELFTEKKYLTSSERQRVANYLDLSDCQVKTWFQNRRTKWKRET

>TLX_Aurelia_sp_complex_Pacific

ISFSINRILRRIGHPYQSRAAPKRKKPRTTFSRSQIAELEELFTEKKYLTSSERQRVANYLSLSDCQVKTWF
QNRRTKWKRET

>TLX_Aurelia_coerulea

ISFSINRILRRIGHPYQSRAAPKRKKPRTTFSRSQIAELEELFTEKKYLTSSERQRVANYLSLSDCQVKTWF
QNRRTKWKRET

>TLX_Sanderia_malayensis

LSFSIKRILRRIGHPYQSRATPKRKKPRTSFTRSQISELEELFTEKKYLTSSERQRVAACLQLTDCQVKTWF
QNRRTKWKRET

>TLX_Chrysaora_achlyos

LSFSIRRILRRIGHPYQSRATPKRKKPRTTFSRSQISELEELFTEKKYLTSSERQRVANYLQLSDCQVKTW
FQNRRTKWKRET

>TLX_Chrysaora_fuscescens

LSFSIRRILRRIGHPYQSRATPKRKKPRTTFSRSQISELEELFTEKKYLTSSERQRVANYLQLSDCQVKTW
FQNRRTKWKRET

>TLX_Cassiopea_xamachana

LSFSISRILRRIGHPYQSRAVPKRKKPRTTFSRAQITELEDLFTEKKYLTSSERQKVASYLNLSDCQVKTWF
QNRRTKWKRET

>TLX_Rhopilema_esculentum

LSFSISRILRRIGHPYQSRAAPKRKKPRTTFSRAQIAELEELFTEKKYLTSSERQKVASYLNLSDCQVKTWF
QNRRTKWKRET

>TLX_Nemopilema_nomurai

LSFSISRILRRVGHPYQSRAAPKRKKPRTTFSRAQIGELEELFTEKKYLTSSERQKVANYLSLSDCQVKTW
FQNRRTKWKRET

>TLX_Lucernaria_quadricornis

ITFSISRILRRVGHPYQSRAPPKRKRPRKAFESDQVEELEHLFHDKRYLASAERQAVARQLGMTDGQVKT
WFQNRRTKWKRQT

>TLX_Calvadosia_cruxmelitensis

ISFSINRILRRIGHPYQSRAPPRRKQPRKSFEADQVAELEQIFHDKRYLTSGERQNVARQLKMTDNQVKT
WFQNRRTKWKRQF

>TLX_Craterolophus_convolvulus

ISFSINRILRRIGHSYQSRALPYRKQPRKTFESRQVDELELLFHTKRYLTSYERQSVAKQLQMSDSQVKTW
FQNRRTKWKRQC

>TLX_Chironex_yamaguchii

LSFSISRILRRLGHPYQSRAPPKRKKQRASFSRHQIKELEKLFSKKKYLSSSERQQIAKKLDMTDCQVKTW
YQNRRTKWKREK

>TLX_Chironex_fleckeri

LSFSISRILRRLGHPYQSRAPPKRKKQRASFSRNQIKELEKLFSKKKYLSSSERQQIAKKLDMTDCQVKTW
YQNRRTKWKREK

>TLX_Alatina_alata

LSFSISRILRRLGHPYQSRAPPKRKKQRASFSRSQIKELEKLFSKKKYLSSSERQQIAKKLDMTDCQVKTW
YQNRRTKWKREK

>TLX_Morbakka_virulenta

LSFSISRILRRLGHPYQSRAPPKRKKQRASFSRGQIKELEKLFSKKKYLSSSERQQIAKNLDMTDCQVKTW
YQNRRTKWKREN

>TLX_Tripedalia_cystophora

LSFSISRILRRLGHPYQSRAPPKRKKQRASFSRSQIKELEKLFSKKKYLSSSERQHIAKKLDMTDCQVKTW
YQNRRTKWKREK

>TLX_Copula_sivicksi

LSFSISRILRRLGHPYQSRAPPKRKKQRASFSRGQIKELEKLFSKKKYLSSSERQQIAKKLEMTDCQVKTW
YQNRRTKWKREK

>TLX_Carybdea_marsupialis

LSFSISRILRRLGHPYQSRAPPKRKKQRASFSRSQIKELEKLFSKKKYLSSSERQQIAKKLDMTDCQVKTW
YQNRRTKWKREK

>TLX_Alatinidae_sp

LSFSISRILRRLGHPYQSRAPPKRKKQRASFSRSQIKELEKLFSKKKYLSSSERQQIAKKLDMTDCQVKTW
YQNRRTKWKREK

>TLX_like_Mnemiopsis_leidyi

LSFSIDQIL-------------
KRKRTRTTFSSAQVYELEKKFQRCQYLSAVDRLNLAAALSMSDVQVKRWFQNRRCKERHRA

>TLX_like_Pleurobrachia_pileus

LSFSIDQIL-------------
KRKRTRTTFSSAQVYELEKKFQRSQYLSAVDRLNLAAALSMSDVQVKRWFQNRRCKERHRA

>HMX_Chrysaora_fuscescence

KKKKTRTVFSRRQVYQLETAFDMKRYLSSSERASLANALKLSETQVKIWFQNRRNKWKRQL

>HMX_Chrysaora_achlyos

KKKKTRTVFSRRQVYQLETAFDMKRYLSSSERASLANALKLSETQVKIWFQNRRNKWKRQL

>HMX_Aurelia_coerulea

KSFRISDIL-------------
KRKKTRTVFSRRQVYELEKAFTLRRYLSSSDRASLATSLKLSETQVKIWFQNRRNKWKRQL

>HMX_Craterolophus_convolvus

KKKKTRTVFSRRQVYQLETAFDVRRYLSSGERSTLARTLQLTETQIKIWFQNRRNKWKRQL

>HMX_Cassiopea_xamachanna

KRKKTRTVFSRRQVYQLESAFELKRYLSSSERASLASSLKLSETQVKIWFQNRRNKWKRQL

>HMX_Nemopilema_nomurai

KRKKTRTVFSRRQVYQLESAFELKRYLSSSERASLASSLKLSETQVKIWFQNRRNKWKRQL

>HMX_Aurelia_aurita

KSFRISDIL-------------
KRKKTRTVFSRRQVYHLETAFERKRYLSSSERASLATSQKLSETQVKIWFQNRRNKWKRQV

>HMX_Craspedacusta_sowerbii

QTFSVNRLL-------------
KKKKNRTVFSRRQVHKLEEWFDHKRYLSSSERAALAQALLLTETQVKIWFQNRRNKWKRQL

>HMX_Morbakka_virulenta

KRKKSRTVFSRRQIYQLESVFDLRRYLSSSERSCLAQELKLSDSQIKIWFQNRRNKLKRQK

>HMX_Tripedalia_cystophora

KSFYITDIL-------------
KKKKSRTVFSRSQVYHLETVFDIRRYLSSTERAYLAQSLNLTEAQIKIWFQNRRNKWKRQV

>HMX_Copula_sivicksii

KKKKSRTVFSRKQIYHLETVFDLHRYLSSTERAYLAQSLDLTEAQIKIWFQNRRNKWKRQI

>HMX_Physalia_physalis

RQKKSRTVFTKKQILKLESMFYAKRYLSNTDRIELSKSLCLSESQVKVWFQNRRNKWKRDM

>HMX_Bargmania_elongata

RQKKSRTVFTKRQILKLESMFYAKRYLSNTDRIELSKSLSLSESQVKVWFQNRRNKWKRDM

>HMX_Clytia_hemisphaerica

FSISSILNL-------------
RKKKSRTVFTRQQIQHLENAFDRKKYFTNSERQKLATDLMLSETQVKIWLQNRRNKWKKQL

>HMX_Hydractinia_symbiolongicarpus

KKKKSRTVFSRYQVEHLENTFKMKRYLTSIERASIAESLTLSETQVKIWFQNRRNKWKRKL

>HMX_Porites_astreoides

TPFSISNIL-------------
RKKKTRTVFSRSQVYQLETTFDLKRYLSSSERAGLAAQLHLTETQVKIWFQNRRNKWKRQL

>HMX_Actinia_tenebrosa

NPFSISNIL-------------
RKKKTRTVFSRSQVYQLESTFDMKRYLSSSERAGLAAQLHLTETQVKIWFQNRRNKWKRQI

>HMX_Ctenactis_echinata

NPFSISNIL-------------
RKKKTRTVFSRSQVYQLESTFDLKRYLSSSERAGLAAQLHLTETQVKIWFQNRRNKWKRQL

>HMX_Anthopleura_elegantissima

NPFSISNIL-------------
RKKKTRTVFSRSQVYQLESTFDMKRYLSSSERAGLAAQLHLTETQVKIWFQNRRNKWKRQI

>HMX_Porpita_porpita

RHKKTRTVFTKQQLVKLESMFFSKRYLNNTDRMELSKTLSLSENQVKVWFQNRRNKWKRDM

>HMX_Vellela_vellela

RHKKTRTVFTKQQLVKLEAMFFSKRYLNNTDRMELSKKLSLSENQVKVWFQNRRNKWKRDL

>HMX_Podocoryna_carnea

KKKKSRTVFSRYQVEHLENTFKMKRYLTSIERASIAESLTLSETQVKIWFQNRRNKWKRKL

>HMX1_Danio_rerio

SSFFIENLL-------------
RKKKTRTVFSRSQVFQLESTFDMKRYLSSSERAGLAASLHLTETQVKIWFQNRRNKWKRQL

>HMX2_Danio_rerio

SSFTIQSIL-------------
AKKKTRTVFSRSQVYQLESTFDMKRYLSSSERACLASSLQLTETQVKTWFQNRRNKWKRQL

>HMX3_Danio_rerio

SPFFIKNLL-------------
RKKKTRTVFSRSQVFQLESTFDMKRYLSSSERAGLAASLHLTETQVKIWFQNRRNKWKRQL

>HMX3_Mus_musculus

SPFSIRNLL-------------
RKKKTRTVFSRSQVFQLESTFDMKRYLSSSERAGLAASLHLTETQVKIWFQNRRNKWKRQL

>HMX2_Mus_musculus

SSFTIQSIL-------------
AKKKTRTVFSRSQVYQLESTFDMKRYLSSSERACLASSLQLTETQVKTWFQNRRNKWKRQL

>HMX1_Mus_musculus

SSFLIENLL-------------
RRKKTRTVFSRSQVFQLESTFDLKRYLSSAERAGLAASLQLTETQVKIWFQNRRNKWKRQL

>HMX1_Homo_sapiens

SSFLIENLL-------------
RKKKTRTVFSRSQVFQLESTFDLKRYLSSAERAGLAASLQLTETQVKIWFQNRRNKWKRQL

>HMX3_Gallus_gallus

ESFYIKNLL-------------
RKKKTRTVFSRSQVFQLESTFDMKRYLSSSERAGLAASLHLTETQVKIWFQNRRNKWKRQL

>HMX1_Gallus_gallus

SSFFIEDLL-------------
RKKKTRTVFSRSQVFQLESTFDVKRYLSSSERAGLAASLHLTETQVKIWFQNRRNKWKRQL

>HMX2_Homo_sapiens

SSFTIQSIL-------------
AKKKTRTVFSRSQVYQLESTFDMKRYLSSSERACLASSLQLTETQVKTWFQNRRNKWKRQL

>HMX3_Xenopus_laevis

SPFSIKSLL-------------
RKKKTRTVFSRSQVFQLESTFDMKRYLSSSERAGLAASLHLTETQVKIWFQNRRNKWKRQL

>HMX_Drosophila_melanogaster

RKKKTRTVFSRAQVFQLESTFDLKRYLSSSERAGLAASLRLTETQVKIWFQNRRNKWKRQL

>NK6_Homo_sapiens

KKHSRPTFSGQQIFALEKTFEQTKYLAGPERARLAYSLGMTESQVKVWFQNRRTKWRKRH

>NK6_Danio_rerio

KKHSRPTFSGQQIFALEKTFEQTKYLAGPERARLAYSLGMTESQVKVWFQNRRTKWRKRH

>NK6_Stylophora_pistillata

RKHTRPTFSGHQIFALEKTFEQTKYLAGPERTRLAYSLGMTESQVKVWFQNRRTKWRKRH

>NK6_Exaiptasia_pallida

RKHTRPTFSGHQIFALEKTFEQTKYLAGPERARLAYSLGMTESQVKVWFQNRRTKWRKRH

>NK6_Copula_sivicksi

KKSSRPTFSGHQIYHLERTFEQTKYLAGPERTRLAHIIGMTENQVKVWFQNRRTKWRKKT

>NK6_Ectopleura_larynx

KSTSRATFKGHQVFHLERVFQKKKYLAGPERASLAKLLMLSENQVKVWFQNRRTKWRKKM

>NK6_Turritopsis_sp_SK_2016

KKQSRPTFNGHQIFHLEKTFESTKYLAGPERSRLAKVLHMSENQVKVWFQNRRTKWRKKL

>NK6_Podocoryna_carnea

KKSSRPTFNGHQIFHLEKTFEKTKYLAGPERGRLAKALRMSENQVKVWFQNRRTKWRKKL

>NK6_Hydractinia_symbiolongicarpus

PKSSRPTFNGHQIFHLEKTFEKTKYLAGPERGRLAKALRMSENQVKVWFQNRRTKWRKKL

>NK6_Rhopilema_esculentum

KKSSRPTFNGRQIYHLEKTFEQTKYLAGPERTRLAHYLAMTENQVKVWFQNRRTKWRKKT

>NK6_Aurelia_sp1

KKSSRPTFNGHQIYHLEKTFEQTKYLAGPERTRLAHYLAMTENQVKVWFQNRRTKWRKKT

>NK6_Clytia_hemisphaerica

NKSSRPTFNGHQIFHLEKTFETTKYLAGPERGRLAKALRMSENQVKVWFQNRRTKWRKKL

>Hex_Homo_sapiens

RKGGQVRFSNDQTIELEKKFETQKYLSPPERKRLAKMLQLSERQVKTWFQNRRAKWRRLK

>Hex_Patiria_miniata

RKGGQVRFSNDQTMELEKKFESQKYLSPPERKKLAKLLQLSERQVKTWFQNRRAKWRRVK

>Hex_Danio_rerio

RKGGQVRFSNDQTIELEKKFETQKYLSPPERKRLAKMLQLSERQVKTWFQNRRAKWRRLK

>Hex_Pocillopora_damicornis

KKGGQVRFSNEQTMELEKIFENQKYLSPPERKQLSKVLGLTERQVKTWFQNRRAKWRRFK

>Hex_Porites_astreoides

KKGGQVRFSNEQTLELEKIFESQKYLSPPERKQLSKVLGLTERQVKTWFQNRRAKWRRFK

>Hex_Nematostella_vectensis

KKGGQVRFSNEQTMELEKIFETQKYLSPPERKQLSKVLGLSERQVKTWFQNRRAKWRRFK

>Hex_Stylophora_pistillata

KKGGQVRFSNEQTMELEKIFENQKYLSPPERKQLSKVLGLTERQVKTWFQNRRAKWRRFK

>Hex_Hydractinia_symbiolongicarpus

RKGGQVRFSHTQSSELERVFSIQKYISPQERKQLSRTLDLTERQVKTWFQNRRAKWRRIK

>Hex_Aurelia_aurita

RKGGQVRFSHVQSVELERIFSIQKYISPQERKQLSRFLHLSERQVKTWFQNRRAKWRRIK

>Hex_Clytia_hemisphaerica

RKGGQVRFTHTQSTELERVFSVQKYVSPQERKQLARSIDLSERQVKTWFQNRRAKWRRIK

>Hex_Podocoryna_carnea

RKGGQVRFSHTQSSELERVFSLQKYISPQERKQLSRTLDLTERQVKTWFQNRRAKWRRIK

>Hex_Xenopus_Laevis

RKGGQVRFSNDQTIELEKKFETQKYLSPPERKRLAKMLQLSERQVKTWFQNRRAKWRRLK

>Hex_Amphiura_filiformis

RKGGQVRFSNDQTLELEKKFENQKYLSPPERKKLAKLLQLSERQVKTWFQNRRAKWRRLK

>Hex_Mus_musculus

RKGGQVRFSNDQTVELEKKFETQKYLSPPERKRLAKMLQLSERQVKTWFQNRRAKWRRLK

Supplementary note 3: Alignment used in Figure S2

>TLX1_Homo_sapiens

ISFGIDQILRRIGHPYQNRTPPKKKKPRTSFTRLQICELEKRFHRQKYLASAERAALAKALKMTD
AQVKTWFQNRRTKWRRQT

>TLX1_Mus_musculus

ISFGIDQILRRIGHPYQNRTPPKKKKPRTSFTRLQICELEKRFHRQKYLASAERAALAKALKMTD
AQVKTWFQNRRTKWRRQT

>TLX1_Gallus_gallus

ISFGIDQILRRIGHPYQNRTPPKKKKPRTSFTRLQICELEKRFHRQKYLASAERAALAKALKMTD
AQVKTWFQNRRTKWRRQT

>TLX1_Alligator_mississippiensis

ISFGIDQILRRIGHPYQNRTPPKKKKPRTSFTRLQICELEKRFHRQKYLASAERAALAKALKMTD
AQVKTWFQNRRTKWRRQT

>TLX1_Danio_rerio

ISFGIDQILRRIGHPYQNRTPPKKKKPRTSFTRLQICELEKRFHRQKYLASAERAALAKALKMTD
AQVKTWFQNRRTKWRRQT

>TLX2_Homo_sapiens

ISFGIDQILRRIGHPYQNRTPPKRKKPRTSFSRSQVLELERRFLRQKYLASAERAALAKALRMTD
AQVKTWFQNRRTKWRRQT

>TLX2_Rattus_norvegicus

ISFGIDQILRRIGHPYQNRTPPKRKKPRTSFSRSQVLELERRFLRQKYLASAERAALAKALRMTD
AQVKTWFQNRRTKWRRQT

>TLX2_Monodelphis_domestica

ISFGIDQILRRVGHPYQNRTPPKRKKPRTSFSRAQVLELERRFLRQKYLASAERAALAKALRMT
DAQVKTWFQNRRTKWRRQT

>TLX2_Sarcophilus_harrisii

ISFGIDQILRRVGHPYQNRTPPKRKKPRTSFSRAQVLELERRFLRQKYLASAERAALAKALRMT
DAQVKTWFQNRRTKWRRQT

>TLX2_Equus_asinus

ISFGIDQILRRIGHPYQNRTPPKRKKPRTSFSRSQVLELERRFLRQKYLASAERAALAKALRMTD
AQVKTWFQNRRTKWRRQT

>TLX3_Homo_sapiens

ISFGIDQILRRIGHPYQNRTPPKRKKPRTSFSRVQICELEKRFHRQKYLASAERAALAKSLKMTD
AQVKTWFQNRRTKWRRQT

>TLX3_Rattus_norvegicus

ISFGIDQILRRIGHPYQNRTPPKRKKPRTSFSRVQICELEKRFHRQKYLASAERAALAKSLKMTD
AQVKTWFQNRRTKWRRQT

>TLX3_Gallus_gallus

ISFGIDQILRRIGHPYQNRTPPKRKKPRTSFSRVQICELEKRFHRQKYLASAERAALAKSLKMTD
AQVKTWFQNRRTKWRRQT

>TLX3_Xenopus_laevis

ISFGIDQILRRIGHPYQNRTPPKRKKPRTSFSRVQICELEKRFHRQKYLASAERAALAKSLKMTD
AQVKTWFQNRRTKWRRQT

>TLX3_Danio_rerio

ISFGIDQILRRIGHPYQNRTPPKRKKPRTSFSRVQICELEKRFHRQKYLASAERAALAKTLKMTD
AQVKTWFQNRRTKWRRQT

>TLX3_Monodelphis_domestica

ISFGIDQILRRIGHPYQNRTPPKRKKPRTSFSRVQICELEKRFHRQKYLASAERAALAKSLKMTD
AQVKTWFQNRRTKWRRQT

>TLX3_Python_bivittatus

ISFGIDQILRRIGHPYQNRTPPKRKKPRTSFSRVQICELEKRFHRQKYLASAERAALAKSLKMTD
AQVKTWFQNRRTKWRRQT

>Clytia_hemisphaerica_Tlx

LSFSIERILRRLGHPYTSRASPKRRAFRHTFTPYQVVQLEKLFEQSRYLSSSERMRMSRELKMT
DNQLKTWYQNRRTKLKREI

>Turritopsis_sp_Tlx

LSFSIENILRRIGHPYKSRAPPKRKPCRNTFTQSQIIQLETLFKKTKYLTSAERLHVAKEINMTDS
QLKTWYQNRRTKLKREI

>Scolionema_suvaense_TLX_from_genome

LKFSIENILRRIGHPYQSRAPPKRKPIRHSFTANQVAELEKLFEKSKYLSSSERQRLASELKMTD
SQLKTWYQNRRTKLKREM

>TLX_Craspedacusta_sowerbii_from_genome_ok

LSFSIDSILRRIGHPYQSRAPPKRKPVRHSFTTSQVAQLEKLFEKSKYLSSADRQRVATQLKMT
DSQLKTWYQNRRTKMKREI

>TLX_Podocoryna_carnea

LSFSIESILRRIGHPYQSRAPPKRKPIRNTFTLPQVQKLEQLFHQTKYLSSAERLRVAKELNMTD
SQLKTWYQNRRTKLKREI

>TLX_Aequorea_australis

LSFSIDSILRRIGHPYKSRAPPKRKPCRKTFTQTQVLQLEKLFEQTRYLSSAERLRMAKELNMTD
SQLKTWYQNRRTKLKREL

>TLX_Laodicea_undulata

ISFSIESILRRIGHPYKSRAPPKRKPCRNTFTQTQVFQLEKLFEQNKYLSSAERLRMAKELNMTD
NQLKTWYQNRRTKLKREM

>TLX_Ptychogena_crocea

ISFSIESILRRIGHPYKSRAPPKRKPCRNTFTPTQVIQMEKLFDQTKYLSSAERLRMAKELDMTDNQLKTWYQNRRTKMKREM

>TLX_Bargmannia_amoena

LSFSIENILRRIGHPYQSRAPPKRKAIRNSFTPNQILHLEKLFEKAKYLPSPERLRVAKELHITDNQLKTWYQNRRTKLKREL

>TLX_Bargmannia_elongata

LSFSIENILRRIGHPYQSRAPPKRKAIRNSFTPNQILHLEKLFEKAKYLPSPERLRVAKELHITDNQLKTWYQNRRTKLKREL

>TLX_Physophora_gilmeri

LSFSIENILRRIGHPYKSRAPPKRKAVRNSFTPGQILRLEKLFEQTKYLPSPERLRVAKELHITDNQLKTWYQNRRTKLKREI

>TLX_Apolemia_lanosa

LSFSIENILRRIGHPYQSRAPPKRKAIRNSFSPNQILQLEKLFEQTKYLPSPERLRVANELHITDNQLKTWYQNRRTKLKREL

>TLX_Apolemia_sp

LSFSIDNILRRIGHPYQSRAPPKRKAIRNSFSPNQILQLEKLFEQTKYLPSPERLRVANELHITDNQLKTWYQNRRTKLKREL

>TLX_Apolemia_rubriversa

LSFSIENILRRIGHPYQSRAPPKRKATRNSFSPNQILQLEKLFQQTKYLPSPERLRVANELHITDNQLKTWYQNRRTKLKREL

>TLX_Forskalia_asymmetrica

LSFSIENILRRIGHPYQSRAPPKRKAVRNSFTPSQVLHLEKLFEQTKYLPSPERLRVAKELHITDNQLKTWYQNRRTKLKREI

>TLX_Lilyopsis_fluoracantha

LSFSIENILRRIGHPYQSRAPPKRKAVRNSFTPSQILHLERLFQQTKYLPSPERLRVAKELRITDNQLKTWYQNRRTKLKREL

>TLX_Atolla_vanhoeffeni

LSFSIHKILRRIGHPYQSRAPPKRKKPRASFTRSQVTELEKLFIKKKYLTSSERQKVAKSLEMSDCQVKTWFQNRRTKWKRET

>TLX_Aurelia_aurita

ITFSIRRILRRIGHPYQSRAAPKRKKPRTTFSRSQIAELEELFTEKKYLTSSERQRVANYLTLSDCQVKTWFQNRRTKWKRET

>TLX_Aurelia_sp1_californian_strain

ISFSINRILRRIGHPYQSRAAPKRKKPRTTFSRSQIAELEELFTEKKYLTSSERQRVANYLSLSDC
QVKTWFQNRRTKWKRET

>TLX_Aurelia_aurita_Kiel_bay_baltic_sea

ISFSISRILRRIGHPYQSRAAPKRKKPRTTFSRSQIAELEELFTEKKYLTSSERQRVANYLDLSDC
QVKTWFQNRRTKWKRET

>TLX_Aurelia_sp_complex_Pacific

ISFSINRILRRIGHPYQSRAAPKRKKPRTTFSRSQIAELEELFTEKKYLTSSERQRVANYLSLSDC
QVKTWFQNRRTKWKRET

>TLX_Aurelia_coerulea

ISFSINRILRRIGHPYQSRAAPKRKKPRTTFSRSQIAELEELFTEKKYLTSSERQRVANYLSLSDC
QVKTWFQNRRTKWKRET

>TLX_Sanderia_malayensis

LSFSIKRILRRIGHPYQSRATPKRKKPRTSFTRSQISELEELFTEKKYLTSSERQRVAACLQLTDC
QVKTWFQNRRTKWKRET

>TLX_Chrysaora_achlyos

LSFSIRRILRRIGHPYQSRATPKRKKPRTTFSRSQISELEELFTEKKYLTSSERQRVANYLQLSD
CQVKTWFQNRRTKWKRET

>TLX_Chrysaora_fuscescens

LSFSIRRILRRIGHPYQSRATPKRKKPRTTFSRSQISELEELFTEKKYLTSSERQRVANYLQLSD
CQVKTWFQNRRTKWKRET

>TLX_Cassiopea_xamachana

LSFSISRILRRIGHPYQSRAVPKRKKPRTTFSRAQITELEDLFTEKKYLTSSERQKVASYLNLSDC
QVKTWFQNRRTKWKRET

>TLX_Rhopilema_esculentum

LSFSISRILRRIGHPYQSRAAPKRKKPRTTFSRAQIAELEELFTEKKYLTSSERQKVASYLNLSDC
QVKTWFQNRRTKWKRET

>TLX_Nemopilema_nomurai

LSFSISRILRRVGHPYQSRAAPKRKKPRTTFSRAQIGELEELFTEKKYLTSSERQKVANYLSLSD
CQVKTWFQNRRTKWKRET

>TLX_Lucernaria_quadricornis

ITFSISRILRRVGHPYQSRAPPKRKRPRKAFESDQVEELEHLFHDKRYLASAERQAVARQLGMT
DGQVKTWFQNRRTKWKRQT

>TLX_Calvadosia_cruxmelitensis

ISFSINRILRRIGHPYQSRAPPRRKQPRKSFEADQVAELEQIFHDKRYLTSGERQNVARQLKMTDNQVKTWFQNRRTKWKRQF

>TLX_Craterolophus_convolvulus

ISFSINRILRRIGHSYQSRALPYRKQPRKTFESRQVDELELLFHTKRYLTSYERQSVAKQLQMSDSQVKTWFQNRRTKWKRQC

>TLX_Chironex_yamaguchii

LSFSISRILRRLGHPYQSRAPPKRKKQRASFSRHQIKELEKLFSKKKYLSSSERQQIAKKLDMTDCQVKTWYQNRRTKWKREK

>TLX_Chironex_fleckeri

LSFSISRILRRLGHPYQSRAPPKRKKQRASFSRNQIKELEKLFSKKKYLSSSERQQIAKKLDMTDCQVKTWYQNRRTKWKREK

>TLX_Alatina_alata

LSFSISRILRRLGHPYQSRAPPKRKKQRASFSRSQIKELEKLFSKKKYLSSSERQQIAKKLDMTDCQVKTWYQNRRTKWKREK

>TLX_Morbakka_virulenta

LSFSISRILRRLGHPYQSRAPPKRKKQRASFSRGQIKELEKLFSKKKYLSSSERQQIAKNLDMTDCQVKTWYQNRRTKWKREN

>TLX_Tripedalia_cystophora

LSFSISRILRRLGHPYQSRAPPKRKKQRASFSRSQIKELEKLFSKKKYLSSSERQHIAKKLDMTDCQVKTWYQNRRTKWKREK

>TLX_Copula_sivicksi

LSFSISRILRRLGHPYQSRAPPKRKKQRASFSRGQIKELEKLFSKKKYLSSSERQQIAKKLEMTDCQVKTWYQNRRTKWKREK

>TLX_Carybdea_marsupialis

LSFSISRILRRLGHPYQSRAPPKRKKQRASFSRSQIKELEKLFSKKKYLSSSERQQIAKKLDMTDCQVKTWYQNRRTKWKREK

>TLX_Alatinidae_sp

LSFSISRILRRLGHPYQSRAPPKRKKQRASFSRSQIKELEKLFSKKKYLSSSERQQIAKKLDMTDCQVKTWYQNRRTKWKREK

>NK6_Homo_sapiens

----------------------KKHSRPTFSGQQIFALEKTFEQTKYLAGPERARLAYSLGMTESQVKVWFQNRRTKWRKRH

>NK6_Danio_rerio

KKHSRPTFSGQQIFALEKTFEQTKYLAGPERARLAYSLGMTESQVKVWFQNRRTKWRKRH

>NK6_Stylophora_pistillata

RKHTRPTFSGHQIFALEKTFEQTKYLAGPERTRLAYSLGMTESQVKVWFQNRRTKWRKRH

>NK6_Exaiptasia_pallida

RKHTRPTFSGHQIFALEKTFEQTKYLAGPERARLAYSLGMTESQVKVWFQNRRTKWRKRH

>NK6_Copula_sivicksi

KKSSRPTFSGHQIYHLERTFEQTKYLAGPERTRLAHIIGMTENQVKVWFQNRRTKWRKKT

>NK6_Ectopleura_larynx

KSTSRATFKGHQVFHLERVFQKKKYLAGPERASLAKLLMLSENQVKVWFQNRRTKWRKKM

>NK6_Turritopsis_sp_SK_2016

KKQSRPTFNGHQIFHLEKTFESTKYLAGPERSRLAKVLHMSENQVKVWFQNRRTKWRKKL

>NK6_Podocoryna_carnea

KKSSRPTFNGHQIFHLEKTFEKTKYLAGPERGRLAKALRMSENQVKVWFQNRRTKWRKKL

>NK6_Hydractinia_symbiolongicarpus

PKSSRPTFNGHQIFHLEKTFEKTKYLAGPERGRLAKALRMSENQVKVWFQNRRTKWRKKL

>NK6_Rhopilema_esculentum

KKSSRPTFNGRQIYHLEKTFEQTKYLAGPERTRLAHYLAMTENQVKVWFQNRRTKWRKKT

>NK6_Aurelia_sp1

KKSSRPTFNGHQIYHLEKTFEQTKYLAGPERTRLAHYLAMTENQVKVWFQNRRTKWRKKT

>NK6_Clytia_hemisphaerica

NKSSRPTFNGHQIFHLEKTFETTKYLAGPERGRLAKALRMSENQVKVWFQNRRTKWRKKL

>Hex_Homo_sapiens

RKGGQVRFSNDQTIELEKKFETQKYLSPPERKRLAKMLQLSERQVKTWFQNRRAKWRRLK

>Hex_Patiria_miniata

RKGGQVRFSNDQTMELEKKFESQKYLSPPERKKLAKLLQLSERQVKTWFQNRRAKWRRVK

>Hex_Danio_rerio

RKGGQVRFSNDQTIELEKKFETQKYLSPPERKRLAKMLQLSERQVKTWFQNRRAKWRRLK

>Hex_Pocillopora_damicornis

KKGGQVRFSNEQTMELEKIFENQKYLSPPERKQLSKVLGLTERQVKTWFQNRRAKWRRFK

>Hex_Porites_astreoides

KKGGQVRFSNEQTLELEKIFESQKYLSPPERKQLSKVLGLTERQVKTWFQNRRAKWRRFK

>Hex_Nematostella_vectensis

KKGGQVRFSNEQTMELEKIFETQKYLSPPERKQLSKVLGLSERQVKTWFQNRRAKWRRFK

>Hex_Stylophora_pistillata

KKGGQVRFSNEQTMELEKIFENQKYLSPPERKQLSKVLGLTERQVKTWFQNRRAKWRRFK

>Hex_Hydractinia_symbiolongicarpus

RKGGQVRFSHTQSSELERVFSIQKYISPQERKQLSRTLDLTERQVKTWFQNRRAKWRRIK

>Hex_Aurelia_aurita

RKGGQVRFSHVQSVELERIFSIQKYISPQERKQLSRFLHLSERQVKTWFQNRRAKWRRIK

>Hex_Clytia_hemisphaerica

RKGGQVRFTHTQSTELERVFSVQKYVSPQERKQLARSIDLSERQVKTWFQNRRAKWRRIK

>Hex_Podocoryna_carnea

RKGGQVRFSHTQSSELERVFSLQKYISPQERKQLSRTLDLTERQVKTWFQNRRAKWRRIK

>Hex_Xenopus_Laevis

RKGGQVRFSNDQTIELEKKFETQKYLSPPERKRLAKMLQLSERQVKTWFQNRRAKWRRLK

>Hex_Amphiura_filiformis

RKGGQVRFSNDQTLELEKKFENQKYLSPPERKKLAKLLQLSERQVKTWFQNRRAKWRRLK

>Hex_Mus_musculus

RKGGQVRFSNDQTVELEKKFETQKYLSPPERKRLAKMLQLSERQVKTWFQNRRAKWRRLK

>Clytia_hemisphaerica_unknown_homeobox

AKKPRTSFSPDQIQALEDSFQEKRYLNHTERMILADELNLTDCQIKTWFQNRRMKMKRQY

>Craspedacusta_sowerbii_uknw_homeobox

PKKPRTSFTPDQIHTLEATFTQKRYLNHTDRMVLADELGLTDCQIKTWFQNRRMKMKRQY

>Podocoryna_carnea_unknown_homeobox

SKKPRTSFTPDQIQTLEDYFNDKHYLNHTERTILAEELNLTDCQIKTWFQNRRMKMKRQY

>Nematostella_vectensis_Tlx_like

KKKPRTAFTESQISELEKRFQSQKYLGSKERSELAGTLGLTDTQVKTWFQNRRMKLKRQR

>ceh-19_like_Actinia_tenebrosa

NKKPRTSFSTEQIRELEKRFETQKYLGTKERAELAISLDLTDTQIKTWFQNRRMKLKRYY

>pnx_Exaiptasia_diaphana

RKKPRTSFTTEQVTALEKRFNSQKYLGTRERSELAEKLNLTDTQIKTWFQNRRMKQKRNV

>Demox_Baikalospongia_intermedia

RKKARTAFSREQVAELEKKFQDKKYLSSAERGELAEKLKLSDMQVKTWFQNRRMKYKRQS

>Demox_Potamolepis_sp._ERM-2005

RKKARTAFSREQVAELEKKFQDKKYLSSAERGELAEKLKLSDMQVKTWFQNRRMKYKRQS

Supplementary note 4: *Tlx* homeodomain codon alignment for RELAX analysis

>TLX_Amphisbetia_minima_degenerate

GCACCACCCAAGCAC---
AAACCAGTCCGAACCACGTTCACGCACGCTCAAGTGTCGCAACTAGAGAACGCCTTCAAACGCAACA
AATATTTGTCACCAGCAGAACGAACACGTATGGCCAAGGATGTTGGTATGACAGACACTCAAATCAA
AACATGG

>TLX_Tiaropsidium_kelseyi_degenerate

GCACCACCAAAACGA---
AAACCGTGCCGAAATACGTTCACCCGATCACAAGTTGTACAACTGGAAAAACTGTTCGAAAGAACCC
GATACCTCTCATCTGCCGAACGATTGCGCATGGCCAAAGAGTTGAACATGACCGACAGTCAGTTAAA
AACATGG

>TLX_Staurocladia_wellingtoni_degenerate

GCACCTCCGAAAAAA---
AAACCTCCAAGAAACACATTTACACAAGCTCAAATAATCCAACTCGAACGAATGTTCGAACGGACTAA
ATATCTCTCATCTAACGAACGTTTACGCATCGCTAAACGCTTGCAAATGACTGACAATCAATTAAAAA
CGTGG

>TLX_Proboscisdactyla_flavicirrata_degenerate

GCACCACCAAAGAAA---
AAACCATCGCGAAATACTTTCACCAAAAGTCAAATATTAAGGTTGGAGAAACTTTTCGAACAGTCAAA
ATACCTATCATCAACGGAACGATTGAGAACTGCAAAAGAACTTGACATGTCAGACAATCAATTGAAAA
CTTGG

>TLX_Leuckartia_octona_degenerate

GCACCACCAAAACGT---
AAACCATGTCGAAATACGTTCACAAATTCTCAAGTAATAAAGCTTGAAACATTATTTCAACAAACAAAA
TATTTACCATCATCAGAACGAATACGTGTGGCCAAGGAATTAAAAATGACAGATAATCAGTTGAAAAC
ATGG

>TLX_Hydrictella_epigorgia_degenerate

GCTCCACCTAAGCGA---
AGACCATCCCGTAACACGTTTACCCAATTCCAGATTATGGAATTAGAAAATTTGTTTCAACAAACCAAA
TATTTATCATCCACTGAACGTTTGCGCATGGCAAAGGAACTGCGTATGACTGATACTCAGCTGAAGA
CTTGG

>TLX_Eleutheria_dichotoma_degenerate

GCCCCTCCAAAACGA---
AAACCTCCTCGCAACACATTCACCCAAGCACAAATCATGCAACTTGAGCGACTATTCGAACGTACGA
AATATCTATCATCAAACGAACGTATCAGAATCGCAAAACGTTTAAAAATGACAGATAACCAAATTAAAA
CCTGG

>TLX_Sarsia_lovenii_degenerate

GCGCCGCCAAAGCAT---
AAAGCACCAAGGAATACGTTCACGCAACTACAGATCAACCAACTGGAGCGATTGTTCGAACGCACAA
AATATCTCTCCTCCTCGGAACGCTTACGTATCTCTAGACGTTTACAAATGACCGATAATCAATTAAAAA
CATGG

>TLX_Polyorchis_haplus_degenerate

GCACCACCCAAAAAT---
AAACCCCCTCGAAACACATTTACGCAACTGCAAATCAATCAGCTGGAACTGTTGTTCGAAAGAACGA
AATATCTGTCTTCATCCGAAAGAATGCGTGTAGCGAAACGTTTACAGATGACAGACAGCCAGTTAAAA
ACATGG

>TLX_Podocoryna_carnea

GCTCCACCGAAAAGA---
AAACCAATTCGTAACACGTTTACACTTCCTCAAGTGCAAAAACTTGAACAATTATTTCATCAAACAAAA
TATTTATCATCGGCAGAGCGATTGCGTGTGGCCAAGGAATTAAATATGACAGACAGTCAGCTCAAAA
CTTGG

>TLX_Turritopsis_sp

GCACCACCAAAACGC---
AAACCTTGTCGCAATACGTTCACACAGTCTCAAATAATCCAACTAGAAACGTTGTTTAAAAAAACAAA
GTATCTAACCTCGGCAGAAAGATTGCATGTGGCCAAGGAAATCAACATGACAGATAGCCAATTAAAA
ACGTGG

>TLX_Aquorea_australis

GCACCACCTAAACGA---
AAACCCTGTAGAAAAACGTTCACACAGACTCAAGTATTACAACTGGAAAAACTATTCGAACAGACGAG
ATATTTATCATCAGCTGAAAGACTTCGTATGGCCAAGGAACTGAATATGACTGATAGTCAATTGAAAA
CCTGG

>TLX_Dynamena_pumila

GCACCTCGAAAACGT---
CAACCGAACCGTACCACGTTCACGCATGCCCAAGTTTTGCAGTTGGAGGACACGTTCAAAACCACAA
AGTATTTGTCGTCAACAGAACGAGCACGTATGGCCAAGGATCTCAGCATGACCAATACTCAAATCAA
AATCTGG

>TLX_Vellela_vellela

GCACCGCCAAAGAGA---
AAACCACCAAGAAATACTTTTACACAACATCAAATATGTGAATTGGAGAGATTATTTGAGCAAGCAAA
ATATCTTTCTTCATCTGAACGATTAAGAGTTGCAAAACGTTTAAACATGACAGATAATCAACTAAAAAC
TTGG

>TLX_Obelia_dichotoma

GCGTCACCAAAACGT---
AAACCATACCGCCACACATTTACGCCCTCACAAGTTATCCAACTCGAAAGTCTATTTGAGAAGAGTCA
ATATCTATCATCGTCCGAACGTGTCCGTGTTGCAAAGGAATTGAAAATGACAGACAACCAAATCAAAA
CATGG

>TLX_Tima_bairdii

GCTCCACCAAAACGT---
AAACCTTGTCGGAACACGTTCACACAAACTCAAGTAGTACAACTAGAAAAACTTTTTGAACAGACCAG
ATATTTATCTTCGGCTGAACGTCTCCGTATGGCCAAGGAATTGAACATGACGGACAATCAGTTGAAAA
CATGG

>TLX_Ptychogena_crocea

GCGCCACCGAAACGC---
AAGCCATGCCGAAACACATTCACTCCAACACAAGTGATTCAGATGGAGAAATTATTTGATCAAACAAA
ATATTTGTCATCGGCAGAACGTTTACGTATGGCCAAGGAACTTGATATGACAGACAACCAATTAAAAA
CGTGG

>TLX_Melicertum_octocostatum

GCTCCACCAAAACGC---
AAAGCAAGTCGAAACTCCTTCACACACTTCCAAGTGGTTGAGCTAGAAAAACTATTTGAAAAAACAAA
GTATTTATCTTCAGCCGAACGCTTACGTACGGCCACGGAACTTCGTATGACTGACAATCAATTGAAAA
CATGG

>TLX_Laodicea_undulata

GCACCACCAAAGCGT---
AAGCCATGTCGAAATACTTTTACTCAAACGCAAGTTTTTCAGCTTGAAAAATTATTCGAACAGAATAAA
TATCTATCCTCCGCAGAACGTTTACGAATGGCTAAAGAACTCAATATGACCGACAACCAATTAAAAAC
ATGG

>TLX_Clytia_islandica

GCATCGCCAAGACGA---
AGGGCGTTCCGCCACACATTCACACCCTATCAAGTCATTCAATTGGAGAAACTGTTTGAACAAAGTC
GTTATTTATCGTCAGCAGAGCGCATGCGTATGTCGAAAGAATTAAAGATTTCGGACAGTCAACTTAAA
ACATGG

>TLX_Clytia_hemisphaerica

GCGTCACCCAAACGA---
AGGGCGTTTCGCCATACATTTACACCCTATCAAGTTGTTCAATTGGAAAAACTATTTGAACAAAGTCG
ATATCTATCGTCATCAGAGCGCATGCGTATGTCACGAGAATTGAAAATGACAGACAACCAATTGAAAA
CGTGG

>TLX_Bargamania_amoena

GCACCTCCGAAAAGG---
AAGGCGATTCGCAATTCGTTTACTCCAAATCAAATTCTGCACTTAGAGAAGCTGTTCGAGAAAGCAAA
GTACCTTCCATCGCCAGAACGTCTGCGTGTGGCGAAGGAACTCCACATAACAGACAATCAACTAAAG
ACGTGG

>TLX_Apolemia_lanosa

GCACCTCCAAAACGA---
AAAGCTATCCGCAATTCTTTTTCTCCGAATCAAATTCTACAGCTTGAGAAACTATTCGAGCAGACAAA
ATATCTTCCGTCACCCGAACGTTTACGAGTGGCAAACGAACTTCATATTACAGATAATCAGCTGAAGA
CGTGG

>TLX_Lyliopsis_fluoracantha

GCACCTCCAAAGCGT---
AAAGCAGTGCGAAATTCTTTTACACCGAGTCAAATATTGCACCTTGAAAGACTTTTTCAGCAAACGAA
GTACTTACCATCTCCAGAAAGACTACGTGTAGCAAAAGAGTTGAGGATTACAGACAATCAACTGAAAA
CGTGG

>TLX_Forskalia_asymmetrica

GCACCACCTAAAAGG---
AAAGCTGTTCGTAATTCATTTACTCCAAGTCAGGTATTACATCTCGAAAAACTATTTGAACAAACTAAG

TACCTTCCATCACCAGAACGCCTACGTGTGGCTAAGGAACTCCATATAACAGATAACCAACTGAAGA
CATGG

>TLX_Erenna_richardi

GCACCACCTAAACGG---
AAAGCAATTCGCAATTCCTTCACTCCAAATCAAATATTACACCTAGAAAAACTCTTTGAGCAAACAAAA
TATCTTCCATCACCTGAACGTTTGCGTGTGGCGAAGGAACTTCATATAACAGATAATCAACTGAAGAC
GTGG

>TLX_Frillagalma_vityazi

GCTCCACCTAAACGG---
AAAGCTGTTCGTAACTCATTCACACCAAGTCAAATATTACACTTAGAAAAACTCTTTGAACAAACAAAA
TATCTTCCATCACCTGAACGTTTGCGTGTGGCCAAGGAACTTCATATAACAGATAATCAACTGAAGAC
GTGG

>TLX_Physophora_gilmeri

GCACCACCAAAAAGA---
AAAGCAGTTCGTAATTCATTCACTCCAGGTCAAATTCTACGCTTAGAAAAACTTTTTGAACAGACAAAA
TATTTACCGTCGCCAGAACGTTTGCGTGTGGCCAAGGAACTTCATATTACCGATAACCAACTGAAGA
CATGG

>TLX_Craspedacusta_sowerbii

GCACCACCCAAACGG---
AAGCCTGTACGTCATTCGTTCACGACGAGTCAAGTAGCTCAACTGGAGAAGCTTTTCGAGAAGTCCA
AATACTTATCCTCGGCAGACCGTCAGCGTGTCGCCACGCAGTTGAAAATGACGGACAGCCAGTTGA
AGACGTGG

>TLX_Cassiopea_xamachana

GCTGTTCCAAAAAGG---
AAAAAAACCAAGAACAACTTTTTCCAGAGCACAGATTACAGAATTGGAGGACTTGTTTACCGAGAAAAA
GTACTTGACCTCTTCAGAACGACAGAAAGTTGCAAGCTACTTGAACTTATCTGACTGCCAAGTGAAAA
CATGG

>TLX_Ectopleura_larynx

GCCCCTGCGAAACGCACGAAGAACGCGCAGAAGATTTTCACCAGGGCGCAGGTGTTTCAACTGGAG
GTTCTGTTTCACGAGAAGAAATATTTGTCATCCACAGAACGTGGCAAGGTGGCAGCTAACCTTCGTA
TGACAGACAGCCAGTTGAAGACGTGG

>TLX_Sanderia_malayenensis

GCGACTCCGAAACGG---
AAAAAGCCCCGTACATCGTTTACGCGATCGCAGATTTCAGAGCTAGAGGAACTGTTCACGGAGAAGA
AGTACTTGACGTCATCGGAGAGGCAACGCGTTGCTGCGTGTCTGCAGCTGACGGACTGTCAAGTGA
AGACCTGG

>TLX_Chrysaora_achlyos

GCAACACCAAAGCGG---
AAAAAACCAAGGACAACATTTTCACGATCCCAAATATCCGAACTCGAGGAACTATTCACAGAGAAAAA
GTACTTGACGTCTTCTGAAAGGCAAAGGGTTGCAAATTATTTACAGCTTTCAGATTGTCAAGTAAAAA
CATGG

>TLX_Chrysaora_quinquecirrha

GCGACGCCTAAACGG---
AAAAAACCGCGCACAACGTTTTCGCGATCTCAGATCCAAGAACTGGAAGAACTGTTTACAGAGAAAA
AATATTTGACGTCATCTGAAAGACAGAGAGTTGCATCTTATCTTCAGCTTACGGACTGTCAAGTAAAA
ACGTGG

>TLX_Chrysaora_chesapeiki

GCGACGCCTAAACGG---
AAAAAACCGCGCACAACGTTTTCGCGATCTCAGATCCAAGAACTGGAAGAACTGTTTACAGAGAAAA
AATATTTGACGTCATCTGAAAGACAGAGAGTTGCATCTTATCTTCAGCTTACGGACTGTCAAGTAAAA
ACGTGG

>TLX_Chrysaora_fuscescence

GCAGCACCAAAGAGA---
AAAAAGCCAAGGACGACGTTTTCCAGAGCACAAATCGGCGAACTGGAAGAATTATTCACGGAGAAAA
AGTACTTGACATCGTCTGAGAGGCAAAAGGTGGCGAATTATCTAAGCCTGTCCGACTGCCAGGTGAA
GACGTGG

>TLX_Aurelia_sp1

GCTGCACCAAAACGA---
AAGAAACCACGAACAACATTTTCAAGATCACAAATTGCAGAATTAGAAGAATTATTCACAGAGAAAAA
ATACCTTACATCATCAGAAAGACAACGAGTTGCAAATTATTTGAGTTTATCAGATTGTCAGGTCAAAAC
ATGG

>TLX_Aurelia_coerulea

GCTGCACCAAAACGA---
AAGAAACCACGAACAACATTTTCAAGATCACAAATTGCAGAATTAGAAGAATTATTCACAGAGAAAAA
ATACCTTACATCATCAGAAAGACAACGAGTTGCAAATTATTTGAGTTTATCAGATTGTCAGGTCAAAAC
ATGG

>TLX_Rhopilema_esculentum

GCAGcaccaaaaaga---
aaaaagccaaGAACGACATTCTCCAGAGCACAAATTGCAGAGCTGGAAGAGCTATTTACagagaaaaaatatc
taaCGTCGTCTGAAAGACAGAAAGTAGCTAGTTATTTGAACTTATCTGATTGCCAGGTAAAGACGTGG

>TLX_Nemopilema_nomurai

GCAGCACCAAAGAGA---
AAAAAGCCAAGGACGACGTTTTCCAGAGCACAAATCGGCGAACTGGAAGAATTATTCACGGAGAAAA
AGTACTTGACATCGTCTGAGAGGCAAAAGGTGGCGAATTATCTAAGCCTGTCCGACTGCCAGGTGAA
GACGTGG

>TLX_Atolla_vanhoeffeni

GCACCTCCAAAAAGA---
AAGAAACCAAGAGCATCATTTACAAGATCACAAGTTACAGAATTAGAAAAACTGTTCATCAAGAAGAA
ATATTTGACTTCATCAGAACGACAAAAGGTCGCGAAATCATTAGAGATGTCAGACTGTCAGGTGAAAA
CCTGG

>TLX_Scolionema_suvaense

GCGCCTCCGAAACGA---

AAACCCATCCGCCATTCGTTCACGGCAAATCAAGTAGCAGAACTTGAGAAGTTATTCGAGAAGTCCA
AGTACCTGTCGTCATCGGAACGTCAGAGACTGGCGTCAGAGCTGAAGATGACAGACAGCCAGCTAA
AGACGTGG

>TLX_Carybdea_marsupialis

GCACCTCCTAAAAGA---
AAGAAGCAACGAGCTTCATTTTCAAGAAGTCAAATCAAAGAActcgaaaaattgttttcaaagaaaaagtatttgtcGTC
TTCCGAACGCCAGCAGATTGCTAAGAAGCTTGACATGACTGACTGTCAAGTAAAAACATGG

>TLX_Chironex_yamaguchii

GCTCCTCCAAAACGG---
AAAAAGCAACGAGCCTCCTTTTCACGACATCAAATCAAAGAACTCGAGAAACTCTTCTCTAAGAAAAA
ATACCTTTCAAGTTCTGAGCGACAGCAGATTGCTAAAAAGCTGGATATGACAGATTGTCAAGTAAAAA
CATGG

>TLX_Chironex_fleckeri

GCTCCTCCAAAACGT---
AAAAAACAGCGAGCCTCCTTTTCACGAAATCAAATCAAAGAACTCGAGAAACTCTTCTCAAAGAAAAA
ATACCTTTCAAGTTCTGAGCGACAGCAGATTGCTAAAAAGCTGGATATGACAGATTGTCAGGTAAAAA
CGTGG

>TLX_Copula_sivscki

GCCCCTCCAAAAAGG---
AAGAAGCAACGCGCATCCTTTTCCCGTGGTCAGATCAAGGAGCTTGAAAAACTGTTTTCCAAAAAGA
AGTACCTCTCTTCGTCTGAACGACAGCAGATCGCCAAAAAGTTGGAGATGACCGACTGCCAAGTAAA
GACATGG

>TLX_Tripedalia_cystophora

GCACCTCCTAAAAGG---
AAAAAGCAGCGTGCCTCCTTTTCCCGGAGCCAGATCAAGGAGTTAGAAAAACTGTTTTCCAAAAAGA
AATACCTCTCTTCGTCTGAAAGACAACATATTGCAAAGAAGCTTGATATGACGGACTGCCAAGTTAAG
ACTTGG

>Tlx_Morbakka_virulenta

GCACctccaaaaagg---
aaaaaacaacgTGCTTCATTTTCCCGTGGTCAAATAAAAGAACTCGAGAAACTGTTCTCCAAGAAAAAGT
ATCTTTCTTCGTCAGAACGGCAGCAAATAGCAAAAAACTTAGACATGACGGACTGCCAAGTCAAAAC
ATGG

>TLX_Lucernaria_quadrecornis

GCGCCGCCAAAACGG---
AAACGCCCGCGCAAGGCATTCGAAAGTGACCAGGTCGAAGAACTGGAACATCTGTTCCACGACAAA
CGCTATCTGGCTTCCGCCGAGCGACAGGCAGTTGCAAGACAACTCGGTATGACGGATGGGCAAGTG
AAAACGTGG

>TLX_Craterolophus_convolvus

GCGCTACCTTATCGC---
AAACAACCGCGCAAGACATTCGAAAGCAGACAAGTGGATGAATTAGAGTTGCTCTTCCACACAAAAC
GATACTTGACTTCATATGAACGGCAAAGTGTTGCTAAGCAACTACAAATGAGTGACAGTCAAGTGAAA
ACGTGG

>TLX_Haliclystus_auricula

GCACCACCAAAAACC---
AAACGGCAACGTCAAGCTTTTAAAAATGATCAAGTTGAAAATCTGGAATCATTATTTTTACAAAAACAT
TATCTTACAACCGAAGAGCGTCGTGCTGTTGCATCTGAATTGCAATTAACAGAACGACAAGTAAAAAC
ATGG

>TLX_Calvadosia_croxementelis

GCACCTCCACGTCGC---
AAACAGCCGCGAAAGTCATTCGAAGCGGACCAAGTAGCCGAATTGGAGCAGATATTTCATGACAAAC
GCTATTTGACATCTGGTGAACGACAGAATGTTGCAAGGCAACTCAAGATGACAGACAACCAAGTTAA
AACGTGG

>TLX_Millepora_squarrosa

GCCCCACCCAAACGC---
AAACCACCACGCAATACATTCACGCAGTTACTTATACGTGAGTTAGAAAGACTGTTTGAGCATACGAA
ATATCTGCCATCACCGGAGAGATTA---
ATTGCCAAAAGATTGCACATAACAGATAATCAATTGAAAACGTGG

>TLX_Drosophila_melanogaster

ACGCCGCCGAAGAGG---
AAAAAGCCACGCACATCCTTCACGCGCATCCAGGTGGCCGAGTTGGAGAAGCGCTTCCACAAGCAA
AAGTATCTGGCATCCGCGGAGCGAGCGGCACTGGCCCGCGGACTGAAGATGACCGATGCCCAGGT
GAAGACGTGG

>TLX_Danio_rerio

ACACCTCCGAAGAAG---
AAGAAGCCCCGGACGTCGTTCACGCGCCTTCAGATATGTGAGCTGGAGAAACGCTTTCACCGTCAG
AAGTATCTGGCGTCAGCCGAGAGAGCAGCCTTAGCGAAAGCACTTAAAATGACTGATGCGCAGGTC
AAAACATGG

>TLX1_Homo_sapiens

ACGCCCCCCAAGAAG---
AAGAAGCCGCGCACGTCCTTCACACGCCTGCAGATCTGCGAGCTGGAGAAGCGCTTCCACCGCCA
GAAGTACCTGGCCTCGGCCGAGCGCGCCGCCCTGGCCAAGGCGCTCAAAATGACCGATGCGCAGG
TCAAAACCTGG

Supplementary figures

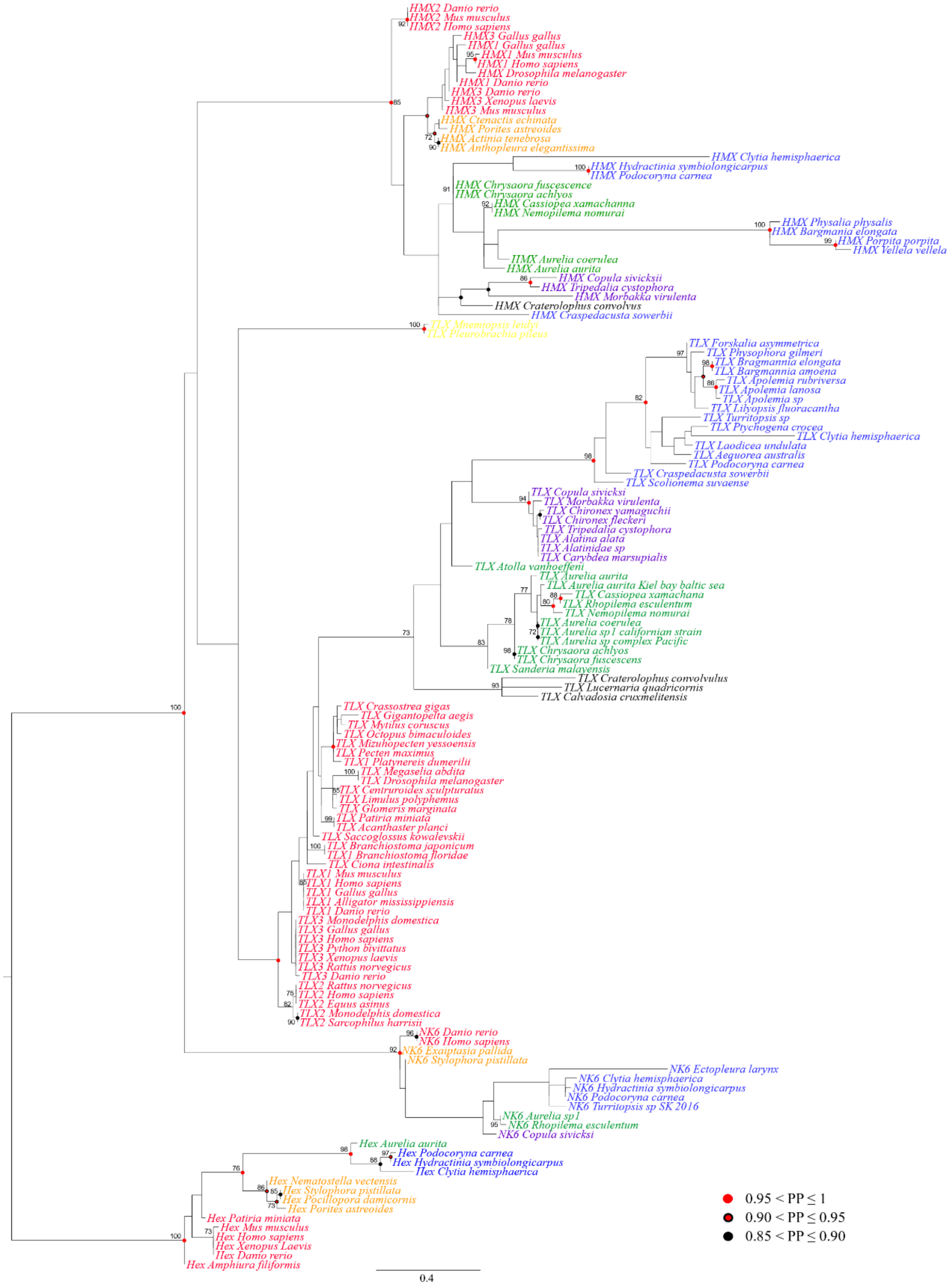

Figure S1. Phylogram from maximum likelihood analysis, with four NK-L representatives (*Tlx*, *Nk6*, *Hmx* and *Hex*). Vertebrate sequences are in red, hydrozoan sequences in blue, staurozoan sequences in black, scyphozoan sequences in green, cubozoan sequences in purple, anthozoan sequences in orange and ctenophore sequences in yellow. Bootstrap values greater than 70% are indicated (1000 bootstraps) next to the nodes. Bayesian posterior probability greater than or equal to 85% are reported on the nodes with colored circles (color code on the figure). Hex sequences are used as the outgroup. Scale bar =

number of inferred substitutions per position in the alignment.

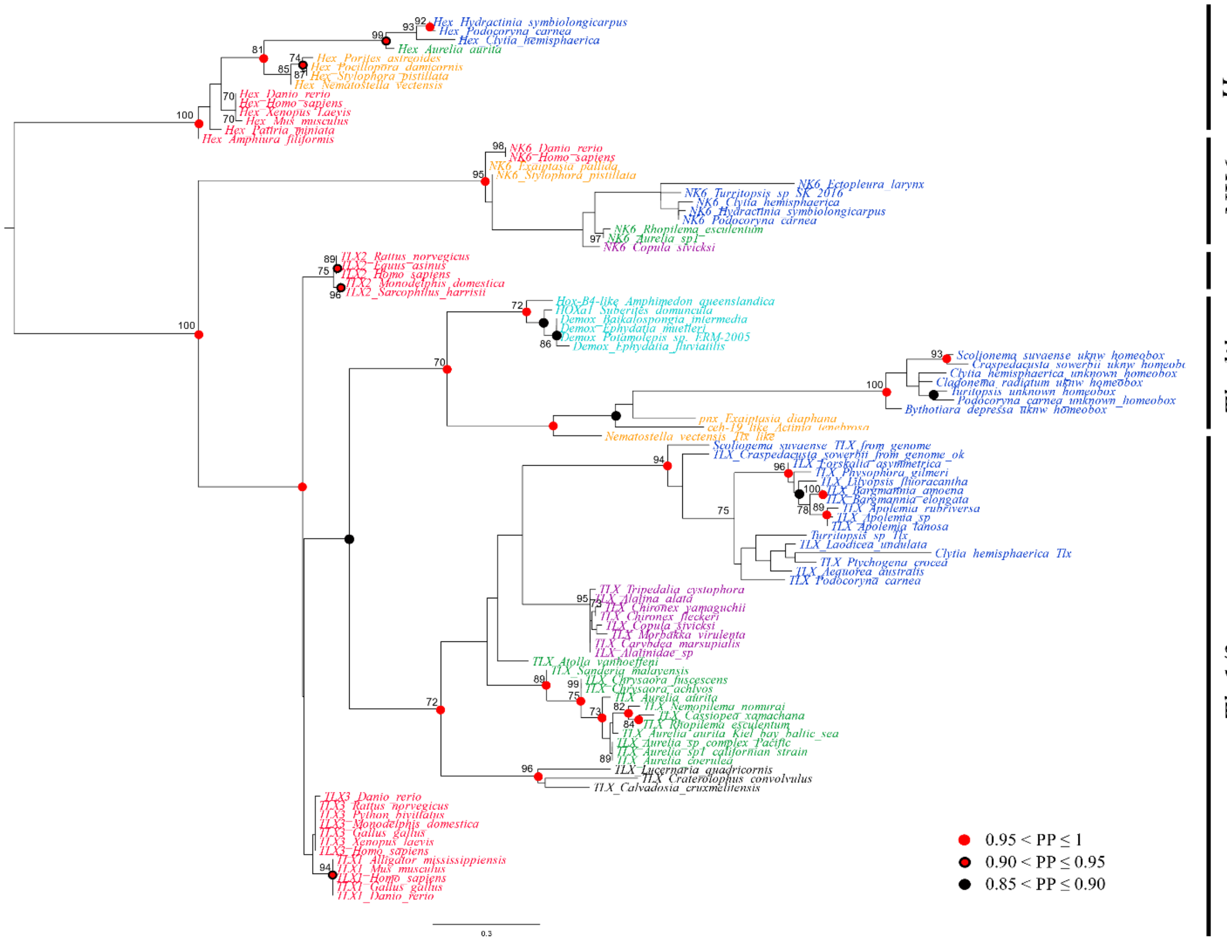

Figure S2. Phylogram from maximum likelihood analysis, with four NK-L representatives (*Tlx*, *Nk6*, *Hex* and an undefined NK-L gene). Vertebrate sequences are in red, sponge sequences in turquoise, hydrozoan sequences in dark blue, staurozoan sequences in black, scyphozoan sequences in green, cubozoan sequences in purple, and anthozoan sequences in orange. Bootstrap values greater than 70% are indicated (1000 bootstraps) next to the nodes. Bayesian posterior probability greater than or equal to 85% are reported on the nodes with colored circles (color code on the figure). Hex sequences are used as the outgroup. Scale bar = number of inferred substitutions per position in the alignment.

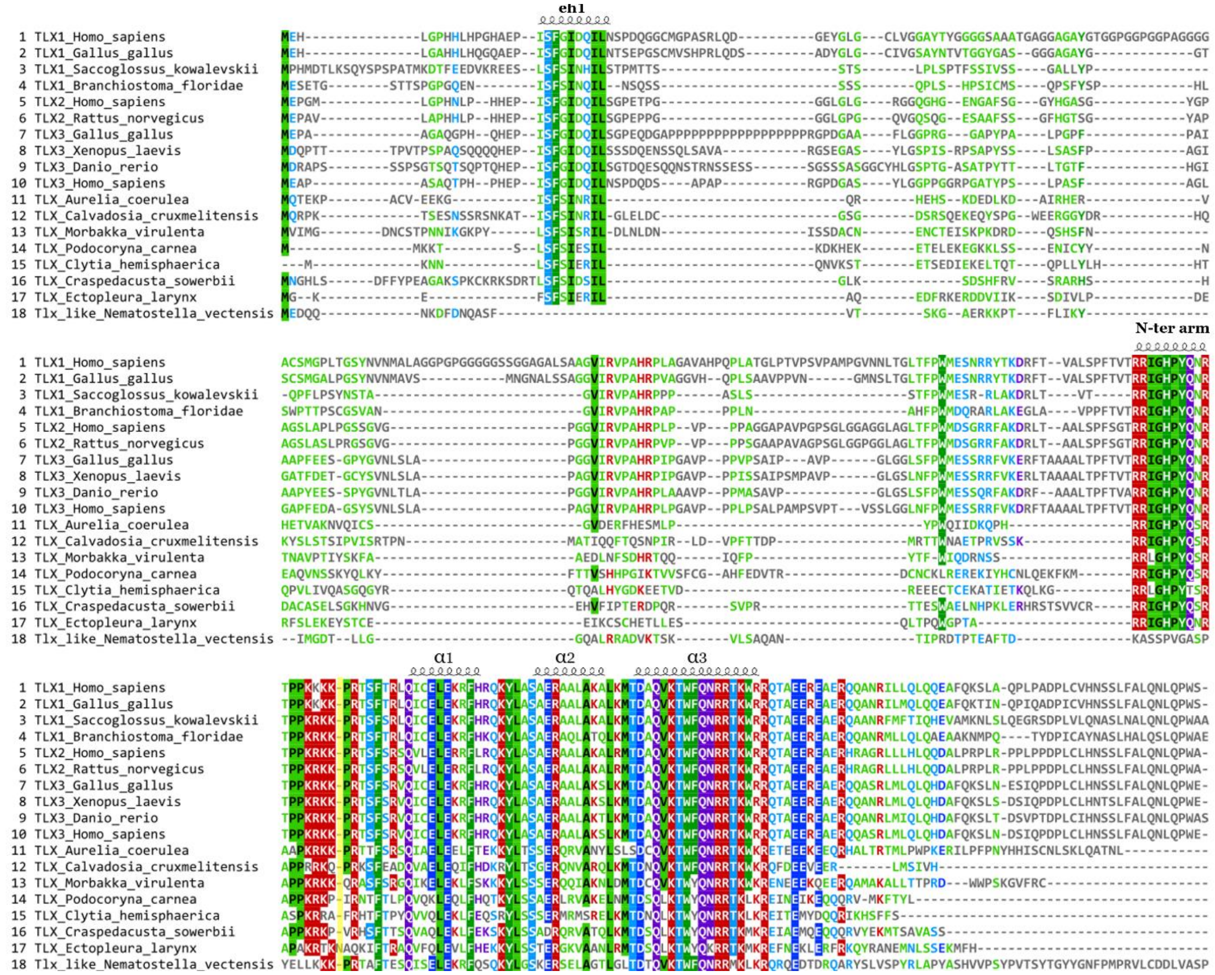

Figure S3. Alignment of complete cnidarian and bilaterian TLX amino acid sequence and the TLX-like sequence from *Nematostella vectensis*. Highly conserved positions are highlighted (>70% identity). Colors represent features of the position, purple (polar uncharged), red (positively charged), blue (negatively charged) and green (hydrophobic). The yellow highlighting indicates the unique asparagine insertion of *Ectopleura larynx.*

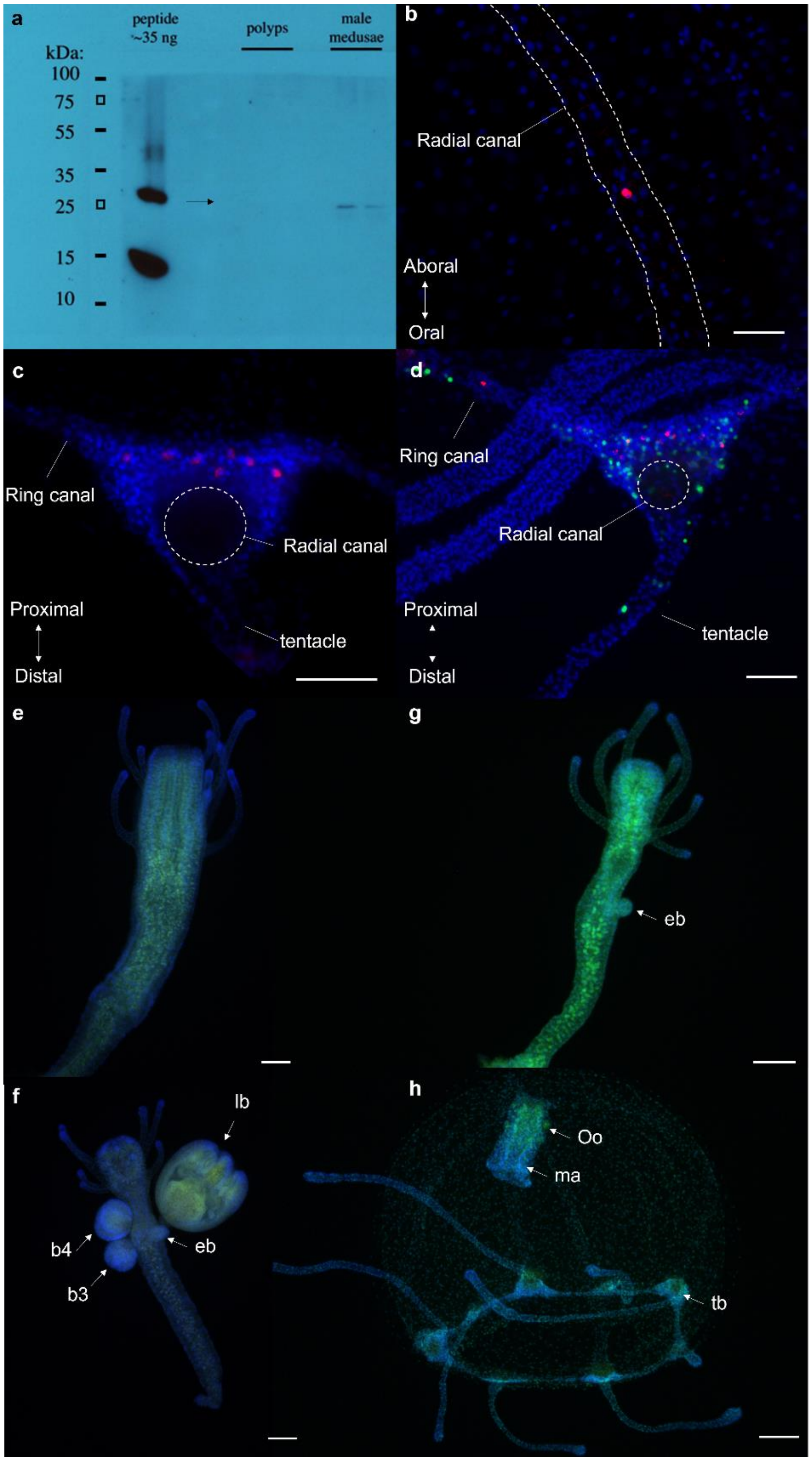

Figure S4. a) Immunoblot validation of the polyclonal antibody targeting PcTLX: protein extracts from non-reproductive polyps and male medusae. *Podocoryna carnea* tissues were analyzed by using custom PcTLX polyclonal antibody PAC 17963-17964. PcTLX atomic mass unit is estimated at 23.18 kDa. The control peptide atomic mass unit is estimated at 13.47kDa. The amount of protein extract loaded was not standardized. b) TLX positive cell (red) in the radial canal of a medusa of *P. carnea*. c)TLX positive

cells arranged in rows (red) in the proximal portion of the tentacle bulb in a medusa of *P. carnea*. d) Tentacle bulb and ring canal of a medusa of *P. carnea* showing TLX positive cells (red) in regions of active cell proliferation (green) and in the ring canals. Medusae of *P. carnea* were incubated in Edu for 24h and Edu labeled DNA was detected using Click-iT Plus EdU Cell Proliferation Kit for Imaging, Alexa Fluor 488 dye (Thermofisher Scientific, C10637). e-h) Immunostaining of histone H1 (green) during the medusa development in *Podocoryna carnea* incubated with Goat anti-Rabbit IgG (H+L) cross-Adsorbed secondary antibody conjugated with Alexa Fluor 594, without PAC 17963-17964 primary antibody incubation. e) non-reproductive, f) early budding polyp, g) budding polyp, h) released female medusa. All samples are counter stained with Hoechst (blue). Abbreviations: b3, medusa bud stage 3; b4 medusa bud stage 4; lb, late bud (stage 5 and above); ma, manubrium; oo, oocyte tb, tentacle bulb. Scale bar: 100 µm (b-d), 200 µm (e-h).

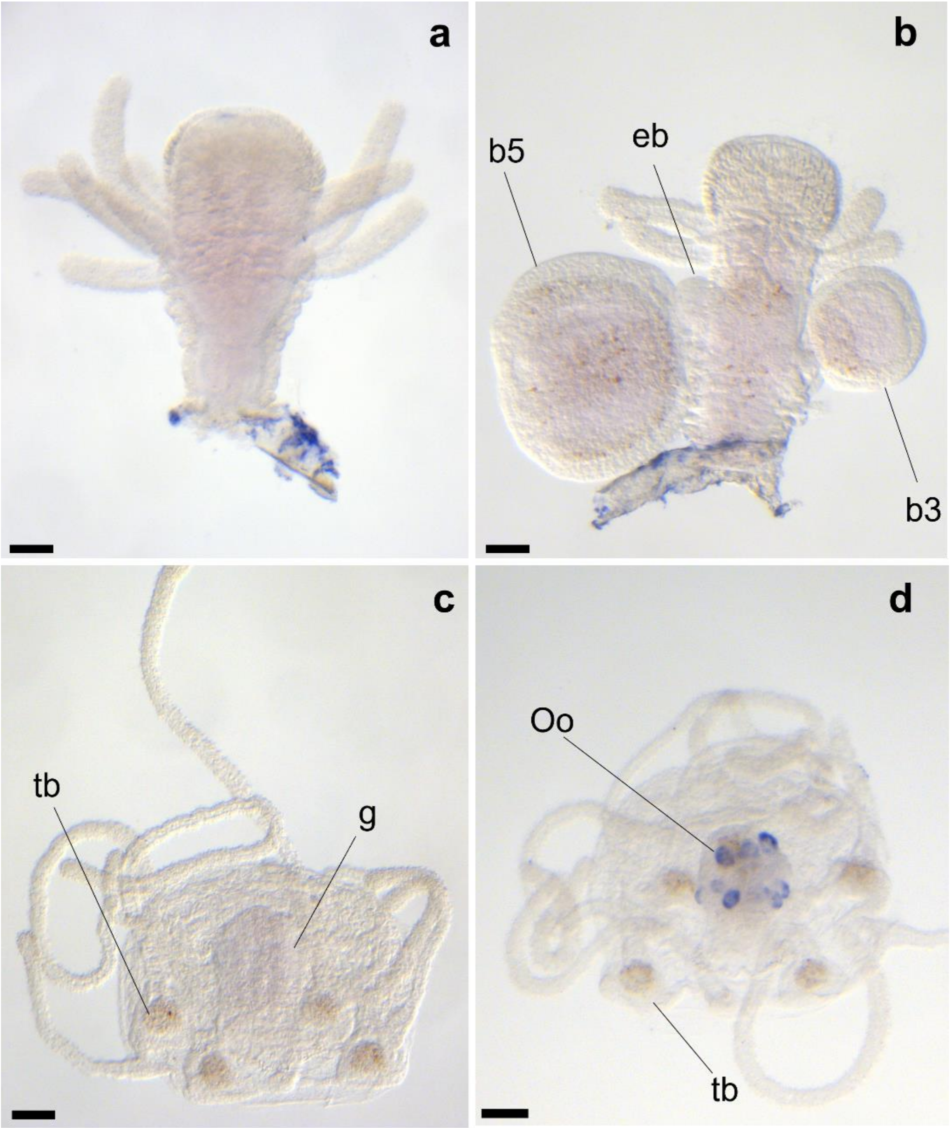

Figure S5. Detection of the sense probes of *PcTlx* on *Podocoryna carnea.* a) in male non-reproductive polyp, b) in a male budding polyp, c) in a male medusa, d) in a female medusa. Samples are presented in radial view, the polyps are presented oral up (a, b) and the medusae oral down (c,d). Abbreviations: : b3,

medusa bud stage 3 b5, medusa bud stage 5; eb, early bud; g, gonad; oo, oocytes; tb, tentacle bulb. Scale bar: 200 µm (a-c), 100 µm (d-j).
