# Supplementary material for: Coevolution of the Tlx homeobox gene with medusa development (Cnidaria: Medusozoa)
Source: Commun Biol. 2023 Jul 11;6:709. doi: 10.1038/s42003-023-05077-6 (PMC10336111; doi:10.1038/s42003-023-05077-6)
Supplement: Supplementary file 3 — Description of Additional Supplementary Files [file 42003_2023_5077_MOESM3_ESM.pdf]

Description of Additional Supplementary Files

File name: Supplementary Data 1
Description: Source of the sequence data, matrices, statistical analyses, numerical values used for graphs in an xls file. This file contains multiple worksheets:'RNA-seq' contains the source of the transcriptome assemblies surveyed for the search of Tlx. 'Genomic data' contains the source of the genome assemblies surveyed for the search of Tlx.'Tlx sequence from GT' contains the Tlx sequences found in this study Page 7 of 17 their source and the taxonomy associated with the samples. 'Tlx degenerate pcr log' contains the source of genomic samples screened for Tlx by PCR, the presence or absence of Tlx amplicon and the source of the genomic samples.'Gene tree accession number' contains the source of the sequences used for the phylogenetic analyses.'Tlx expression data' contains the numerical value for the differential expression analyses, the RTqPCR and the output from the statistical tests. 'Bayestraits matrix' contains the coding of the character used to perform the Bayesian correlation analysis with bayestraits.
