# Supplementary material for: Coevolution of the Tlx homeobox gene with medusa development (Cnidaria: Medusozoa)
Source: Commun Biol. 2023 Jul 11;6:709. doi: 10.1038/s42003-023-05077-6 (PMC10336111; doi:10.1038/s42003-023-05077-6)
Supplement: Supplementary file 5 — Reporting Summary [file 42003_2023_5077_MOESM5_ESM.pdf]

nature portfolio

Corresponding author(s): Matthew Travert

Last updated by author(s): Jun 16, 2023

Reporting Summary

Nature Portfolio wishes to improve the reproducibility of the work that we publish. This form provides structure for consistency and transparency in reporting. For further information on Nature Portfolio policies, see our Editorial Policies and the Editorial Policy Checklist.

Statistics

For all statistical analyses, confirm that the following items are present in the figure legend, table legend, main text, or Methods section.

n/a	Confirmed	
☐	☒	The exact sample size (*n*) for each experimental group/condition, given as a discrete number and unit of measurement
☐	☒	A statement on whether measurements were taken from distinct samples or whether the same sample was measured repeatedly
☐	☒	The statistical test(s) used AND whether they are one- or two-sided<br>*Only common tests should be described solely by name; describe more complex techniques in the Methods section.*
☒	☐	A description of all covariates tested
☒	☐	A description of any assumptions or corrections, such as tests of normality and adjustment for multiple comparisons
☐	☒	A full description of the statistical parameters including central tendency (e.g. means) or other basic estimates (e.g. regression coefficient) AND variation (e.g. standard deviation) or associated estimates of uncertainty (e.g. confidence intervals)
☐	☒	For null hypothesis testing, the test statistic (e.g. *F*, *t*, *r*) with confidence intervals, effect sizes, degrees of freedom and *P* value noted<br>*Give P values as exact values whenever suitable.*
☒	☐	For Bayesian analysis, information on the choice of priors and Markov chain Monte Carlo settings
☒	☐	For hierarchical and complex designs, identification of the appropriate level for tests and full reporting of outcomes
☒	☐	Estimates of effect sizes (e.g. Cohen's *d*, Pearson's *r*), indicating how they were calculated

Our web collection on statistics for biologists contains articles on many of the points above.

Software and code

Policy information about availability of computer code

Data collection	Camera software, InfinityAnalyze v6.5.
Data analysis	Alignment performed using MEGA7, phylogenetic analyses using Mrbayes 3.2.7 and RaxML-HPC2 on XSEDE, characters ancestral state recontruction using Mesquite v3.61, protein model test using Modeltest-NG, traits correlation analysis using BayesTraits v3, reads trimming using fastp, transcriptome assembly using Trinity v2.8.5, genome assembly using Spades v3.13.1, read mapping on genome using STAR 2.7.10b, gene expression quantification using RSEM v1.3.2, differential gene expression analysis using EBseq 1.38.0.Imnustaining pictures processing in ImageJ v1.53e

For manuscripts utilizing custom algorithms or software that are central to the research but not yet described in published literature, software must be made available to editors and reviewers. We strongly encourage code deposition in a community repository (e.g. GitHub). See the Nature Portfolio guidelines for submitting code & software for further information.

Data

Policy information about availability of data

All manuscripts must include a data availability statement. This statement should provide the following information, where applicable:

- Accession codes, unique identifiers, or web links for publicly available datasets
- A description of any restrictions on data availability
- For clinical datasets or third party data, please ensure that the statement adheres to our policy

All data supporting the findings of this study are available within the paper and its Supplementary Information. The transcriptome of Podocoryna carnea and associated reads have been deposited in Genbank (BioProject ID PRJNA245897). The accession numbers of the sequences used in this study are provided in the Supplementary Table S1. The accession number or links of the genomes and transcriptomes assembly used in this study are provided in the Supplementary Table S1. When genomes or transcriptomes were assembled from reads the SRA accession number are provided in the Supplementary table S1.

Research involving human participants, their data, or biological material

Policy information about studies with human participants or human data. See also policy information about sex, gender (identity/presentation), and sexual orientation and race, ethnicity and racism.

Reporting on sex and gender	N/A
Reporting on race, ethnicity, or other socially relevant groupings	N/A
Population characteristics	N/A
Recruitment	N/A
Ethics oversight	N/A

Note that full information on the approval of the study protocol must also be provided in the manuscript.

Field-specific reporting

Please select the one below that is the best fit for your research. If you are not sure, read the appropriate sections before making your selection.

☐ Life sciences ☐ Behavioural & social sciences ☒ Ecological, evolutionary & environmental sciences

For a reference copy of the document with all sections, see nature.com/documents/nr-reporting-summary-flat.pdf

Ecological, evolutionary & environmental sciences study design

All studies must disclose on these points even when the disclosure is negative.

Study description	Correlation analysis of the phylogenetic distribution of a genetic trait (Tlx gene) with a developmental trait (medusa type)<br>Differential expression of Tlx across distantly related species during the medusa development (Podocoryna carnea, Aurelia coerulea and Clytia hemisphaerica)<br>Selection analyses on the Tlx sequence in relation to the medusa type<br>Characterization of the gene expression pattern of Tlx mRNA through in situ hybridization in two distantly related species (Podocoryna carnea and Pelagia noctiluca)<br>Characterization of the gene expression pattern of the TLX protein in Podocoryna carnea
Research sample	Podocoryna carnea (Medusozoa, Hydrozoa, Hydractiniidae). Female (PCLH001), Male (PCLH002), male F1 (PCLH001 X PCLH002). P.carnea (PCLH001 and PCLH002) collected in Connecticut,USA and are maintained in the lab at the Univeristy of Kansas.<br>The list of genomic DNA samples and the voucher information of the cnidarians surveyed are provided in the supplements Table S1<br>Pelagia noctiluca(Medusozoa, Scyphozoa,Pelagiidae), collected in Villefranche-sur-mer, France and are maintained in the Lab at the Villefranche-sur-Mer Marine Station
Sampling strategy	N/A
Data collection	Matthew Travert collected Podocoryna carnea samples, imaged results from ISH and IHC on P.carnea, Steve Sanders imaged the WB validation of the antibody, Lucas Leclere and Manon Boosten imaged the ISH on P.noctiluca.
Timing and spatial scale	N/A

Data exclusions	No data were excluded
Reproducibility	In situ hybridization and immunostaining experiments were repeated and all attempts were successful
Randomization	N/A
Blinding	Blinding was not possible as the results were obvious from the images.

Did the study involve field work? ☐ Yes ☒ No

Reporting for specific materials, systems and methods

We require information from authors about some types of materials, experimental systems and methods used in many studies. Here, indicate whether each material, system or method listed is relevant to your study. If you are not sure if a list item applies to your research, read the appropriate section before selecting a response.

Materials & experimental systems

n/a	Involved in the study
☐	☒ Antibodies
☐	☐ Eukaryotic cell lines
☐	☐ Palaeontology and archaeology
☐	☒ Animals and other organisms
☐	☐ Clinical data
☐	☐ Dual use research of concern
☐	☐ Plants

Methods

n/a	Involved in the study
☐	☐ ChIP-seq
☐	☐ Flow cytometry
☐	☐ MRI-based neuroimaging

Antibodies

Antibodies used	Custom polyclonal antibody targeting Podocoryna carnea TLX produced in rabbit, generated by Pacific immunology<br>Mouse anti-Histone H1 (Leinco Technologies Cat#561 H126, RRID:AB_2830309)<br>Goat Anti-Mouse IgG (H+L) secondary 563 antibody conjugated with CF488A (Sigma-Aldrich Cat# SAB4600042, RRID:AB_2532075)<br>Goat anti-<br>564 Rabbit IgG (H+L) cross-Adsorbed secondary antibody conjugated with Alexa Fluor 594 (Thermo Fisher 565 Scientific Cat# A-11012, RRID:AB_2534079)
Validation	Primary antibodies were validated through western blot on the protein extract of Podocoryna carnea. The primary antibody was produced in rabbit and affinity purified (target peptidKLH carrier protein).

Eukaryotic cell lines

Policy information about cell lines and Sex and Gender in Research

Cell line source(s)	N/A
Authentication	N/A
Mycoplasma contamination	N/A
Commonly misidentified lines (See ICLAC register)	N/A

Palaeontology and Archaeology

Specimen provenance	N/A
Specimen deposition	N/A
Dating methods	N/A

☐ Tick this box to confirm that the raw and calibrated dates are available in the paper or in Supplementary Information.

Ethics oversight	N/A

Note that full information on the approval of the study protocol must also be provided in the manuscript.

Animals and other research organisms

Policy information about studies involving animals; ARRIVE guidelines recommended for reporting animal research, and Sex and Gender in Research

Laboratory animals	Podocoryna carnea (Medusozoa, Hydrozoa, Hydractiniidae). Female (PCLH001), Male (PCLH002), male F1 (PCLH001 X PCLH002). P.carnea (PCLH001 and PCLH002) collected in Connecticut,USA and are maintained in the lab at the Univeristy of Kansas. Pelagia noctiluca(Medusozoa, Scyphozoa,Pelagiidae), collected in Villefranche-sur-mer, France and are maintained in the Lab at the Villefranche-sur-Mer Marine Station
Wild animals	N/A
Reporting on sex	For Podocoryna carnea both male and females were used as this study address both somatic and germ line development
Field-collected samples	N/A
Ethics oversight	No ethical approval was required as the animals used in this study are marine invertebrates that are not subject to any ethical regulation

Note that full information on the approval of the study protocol must also be provided in the manuscript.

Clinical data

Policy information about clinical studies
All manuscripts should comply with the ICMJE guidelines for publication of clinical research and a completed CONSORT checklist must be included with all submissions.

Clinical trial registration	N/A
Study protocol	N/A
Data collection	N/A
Outcomes	N/A

Dual use research of concern

Policy information about dual use research of concern

Hazards

Could the accidental, deliberate or reckless misuse of agents or technologies generated in the work, or the application of information presented in the manuscript, pose a threat to:

No	Yes	
☒	☐	Public health
☒	☐	National security
☒	☐	Crops and/or livestock
☒	☐	Ecosystems
☒	☐	Any other significant area

Experiments of concern

Does the work involve any of these experiments of concern:

No	Yes	
☒	☐	Demonstrate how to render a vaccine ineffective
☒	☐	Confer resistance to therapeutically useful antibiotics or antiviral agents
☒	☐	Enhance the virulence of a pathogen or render a nonpathogen virulent
☒	☐	Increase transmissibility of a pathogen
☒	☐	Alter the host range of a pathogen
☒	☐	Enable evasion of diagnostic/detection modalities
☒	☐	Enable the weaponization of a biological agent or toxin
☒	☐	Any other potentially harmful combination of experiments and agents

Plants

Seed stocks	N/A
Novel plant genotypes	N/A
Authentication	N/A

ChIP-seq

Data deposition

☐ Confirm that both raw and final processed data have been deposited in a public database such as GEO.

☐ Confirm that you have deposited or provided access to graph files (e.g. BED files) for the called peaks.

Data access links<br>*May remain private before publication.*	N/A
Files in database submission	N/A
Genome browser session<br>(e.g. UCSC)	N/A

Methodology

Replicates	N/A
Sequencing depth	N/A
Antibodies	N/A
Peak calling parameters	N/A
Data quality	N/A
Software	N/A

Flow Cytometry

Plots

Confirm that:

☐ The axis labels state the marker and fluorochrome used (e.g. CD4-FITC).

☐ The axis scales are clearly visible. Include numbers along axes only for bottom left plot of group (a 'group' is an analysis of identical markers).

☐ All plots are contour plots with outliers or pseudocolor plots.

☐ A numerical value for number of cells or percentage (with statistics) is provided.

Methodology

Sample preparation	N/A
Instrument	N/A
Software	N/A
Cell population abundance	N/A
Gating strategy	N/A

☐ Tick this box to confirm that a figure exemplifying the gating strategy is provided in the Supplementary Information.

Magnetic resonance imaging

Experimental design

Design type	N/A
Design specifications	N/A
Behavioral performance measures	N/A

Acquisition

Imaging type(s)	N/A
Field strength	N/A
Sequence & imaging parameters	N/A
Area of acquisition	N/A

Diabetes MRI ☐ Used ☐ Not used

Preprocessing

Preprocessing software	N/A
Normalization	N/A
Normalization template	N/A
Noise and artifact removal	N/A
Volume censoring	N/A

Statistical modeling & inference

Model type and settings	N/A
Effect(s) tested	N/A

Specify type of analysis: ☐ Whole brain ☐ ROI-based ☐ Both

Statistic type for inference (See Eklund et al. 2016)	N/A
Correction	N/A

Models & analysis

n/a	Involved in the study
☒	☐ Functional and/or effective connectivity
☒	☐ Graph analysis
☒	☐ Multivariate modeling or predictive analysis
